# *O*-GlcNAcylation of XRCC4 controls its stability and confers resistance to DNA double-strand break damage in cancer cells

**DOI:** 10.1038/s41419-025-08209-4

**Published:** 2026-01-09

**Authors:** Jeong Yeon Ko, Tae Hyun Kweon, Hyeryeon Jung, Jingu Kang, Yeolhoe Kim, Yun Ju Kim, Donghyuk Shin, Seong Wook Yang, Myeong Min Lee, Jun Young Hong, Jae-Min Lim, Eugene C. Yi, Jin Won Cho, Won Ho Yang

**Affiliations:** 1 Department of Systems Biology, College of Life Science and Biotechnology, Yonsei University, 50 Yonsei-ro, Seodaemun-gu, Seoul, 03722 Republic of Korea; 2 Interdisciplinary Program of Integrated OMICS for Biomedical Science, Graduate School, Yonsei University, 03722 Seoul, Republic of Korea; 3 Department of Molecular Medicine and Biopharmaceutical Sciences, School of Convergence Science and Technology and College of Medicine or College of Pharmacy, Seoul National University, 28 Yeongeon-dong, Jongno-gu, Seoul, 03080 Republic of Korea; 4 Department of Chemistry, College of Natural Sciences, Changwon National University, Changwon, 51140 Republic of Korea; 5 Glycosylation Network Research Center, Yonsei University, 50 Yonsei-ro, Seodaemun-gu, Seoul, 03722 Republic of Korea

**Keywords:** Double-strand DNA breaks, Oncogenes, Non-homologous-end joining, Glycosylation

## Abstract

X-ray repair cross-complementing protein 4 (XRCC4), a non-homologous end-joining protein involved in DNA double-strand break repair, is highly expressed in human cancer cells and tissues. A prior OGT interactome study identified XRCC4 as a candidate for *O*-GlcNAcylation. *O*-GlcNAcylation levels, a post-translational modification found on nuclear and cytosolic proteins, are also elevated in various cancers. However, the direct regulatory mechanism linking *O*-GlcNAcylation to XRCC4 function in cancer cells remains unclear. Here, we found that XRCC4 is *O*-GlcNAcylated at threonine 308, enhancing its stability by inhibiting TRIM21-mediated ubiquitin-dependent proteasomal degradation. *O*-GlcNAcylation elevated XRCC4 protein levels during DNA double-strand break damage, thereby conferring resistance to such damage. Additionally, XRCC4 Thr308 *O*-GlcNAcylation promotes cancer proliferation, invasion, and in vivo tumor growth. These findings suggest that downregulating *O*-GlcNAcylation on XRCC4 could be a potential therapeutic strategy to increase cancer sensitivity to chemotherapy or radiotherapy.

## Introduction

Genomic instability drives tumorigenesis; however, it also represents an intrinsic vulnerability of cancer cells [1]. Chemotherapy, as well as hypoxic and nutrient-deprived tumor microenvironments, exacerbates DNA breakage [2, 3]. Accumulated DNA damage activates the DNA damage response (DDR), which leads to cell death or senescence, halting cancer growth [4, 5]. Cancer cells primarily repair DNA double-strand breaks (DSBs) via non-homologous end-joining (NHEJ) and homologous recombination (HR) [6]. In mammalian cells, NHEJ is the main pathway for DSB repair, especially for ionizing radiation (IR)-induced DSBs, throughout the cell cycle [7, 8]. X-ray repair cross-complementing protein 4 (XRCC4) is a key component of the NHEJ pathway, which plays a crucial role in chemoresistance and cancer cell survival [6, 9]. The XRCC4-DNA ligase IV (LIG4) complex is essential for this process as it ligates broken DNA ends, thereby completing DSB repair [10]. Without XRCC4, binding of LIG4 to DNA is challenging, thereby hindering DNA DSB repair [11]. Moreover, XRCC4 regulates LIG4 stability, whose expression is undetectable in XRCC4 knockout cells [12, 13]. Consequently, the absence of XRCC4 jeopardizes cancer cell survival and proliferation due to impaired DNA DSB repair. On the other hand, an earlier study of the OGT interactome revealed XRCC4 as a target of *O*-GlcNAcylation [14]. While XRCC4 has been shown to be modulated by phosphorylation or SUMOylation, the role of *O*-GlcNAcylation on XRCC4 has not been explored [15–18].

*O*-GlcNAcylation is a post-translational modification that regulates fundamental cellular processes, including metabolism [19, 20], neurodegenerative diseases [21, 22], and cancer [23–25]. This modification is dynamically controlled by two enzymes: *O*-GlcNAc transferase (OGT) and *O*-GlcNAcase (OGA). These enzymes add and remove *O*-GlcNAc from serine (Ser) and threonine (Thr) residues of several nuclear and cytoplasmic proteins, respectively. Uridine diphosphate GlcNAc (UDP-GlcNAc), the precursor of *O*-GlcNAc, is synthesized via the hexosamine biosynthetic pathway (HBP) from 2 to 5% of the total glucose uptake [26]. *O*-GlcNAcylation is extremely sensitive to nutrient availability, considering that it is a glucose-derived precursor. Additionally, recent studies have revealed that *O*-GlcNAcylation is also responsive to various cellular stresses like heat shock and hypoxia [27, 28]. Thus, *O*-GlcNAcylation acts as a nutrient and stress sensor that modulates diverse cellular processes.

Previous research has shown that *O*-GlcNAcylation affects several tumor-associated proteins, such as p53, c-Myc, and beta-catenin [29, 30]. Abnormal *O*-GlcNAc levels and OGT and OGA expression have been observed in several cancer models, including cell lines and human tumor samples [25]. *O*-GlcNAcylation is associated with tumor prognosis and tumor grade in humans [31, 32]. Additionally, elevated XRCC4 levels have been found in human glioblastoma, lung cancer, and breast cancer cell lines [33, 34]. However, the precise regulatory mechanism linking *O*-GlcNAcylation to XRCC4 function in cancer cells remains to be determined. Here, our study reveals that *O*-GlcNAcylation of XRCC4 at Thr308 enhances its stability by inhibiting TRIM21-mediated K48-ubiquitin-dependent proteasomal degradation. This mechanism confers resistance to DSB damage in cancer cells. These findings suggest a significant relationship between cellular *O*-GlcNAcylation and DNA DSB repair, highlighting the role of *O*-GlcNAcylation in promoting DNA DSB repair through XRCC4 expression modulation.

## Materials and methods

### Cell culture and reagents

HEK293, MCF7, HCT116, HCT116 XRCC4 knockout cell line, and each of the established stable cell lines were all cultured in Dulbecco’s Modified Eagle’s Medium (Welgene, #LM 001-05, South Korea) supplemented with 10% fetal bovine serum (FBS) (Gibco, #16000-044, USA), 100 U/mL penicillin and 100 μg/mL streptomycin (Gibco, #15140122, USA) at 37 °C in 5% CO_2_. A549 cells were cultured in RPMI-1640 Medium (Hyclone, #SH30255.01, USA) at 37 °C in 5% CO_2_. MCF-10A cells were cultured in DMEM/F12 (Gibco, #11320-033) supplemented with 5% Horse serum (Gibco, #16050122), 10 μg/mL human Insulin (Sigma, #I2643), 20 ng/mL human EGF (PeproTech INC., #AF-100-15), 500 ng/mL Hydrocortisone (Sigma, #H0396), and 100 U/mL penicillin and 100 μg/mL streptomycin (Gibco, #15140122, USA) at 37 °C in 5% CO_2_. HCT116, A549, CCD-33Co, CCD-112CoN, HT29, DLD1, MCF-10A, MCF7, MDA-MB-231, and MDA-MB-468 cells were obtained from the American Type Culture Collection. HCC-1588, NCI-H460, NCI-H69, IMR-90, and MRC-5 cells were obtained from the Korean Cell Line Bank. All cells were tested for mycoplasma contamination using a mycoplasma detection polymerase chain reaction (PCR) test (Bionics, South Korea). Thiamet-G (#SML0244-25MG), OSMI-1 (#SML1621-25MG), cycloheximide (#C7698), and MG132 (#474790) were purchased from Sigma-Aldrich (St Louis, MO, USA). Bleomycin (#13877) was purchased from Cayman Chemical.

### Transfection and plasmids

Transfection was performed using either polyethylenimine (PEI) or Omicsfect (#CP2101) according to the manufacturer’s protocol. XRCC4 was prepared by PCR and subcloned into the EcoRI and BamHI sites of the p3XFLAG-CMV7.1 vector (Sigma-Aldrich, USA). XRCC4 point mutants were generated using Muta-Direct Site-Directed Mutagenesis Kit (iNtRON, #15071, South Korea) according to the manufacturer’s protocol. Quality check for the mutagenesis was conducted by DNA sequencing (Bionics, South Korea). TRIM21 was cloned into the XhoI and HindIII sites of the pcDNA3.1-mycHis (−) A vector (Invitrogen, USA). pMSCV-hygro/FLAG vector was kindly provided by Prof. Jaewhan Song (Yonsei University, Seoul, South Korea). siRNA targeting TRIM28 or TRIM21 transfection was performed using Lipofectamine RNAiMAX (Thermo Fisher Scientific, Pittsburgh, PA, USA) following the manufacturer’s protocol. The siRNA sequences were: siControl (duplex, Bioneer, #SN-1002, Korea), siTRIM28 (Bioneer, #SDO-1001, Korea), siTRIM21 (sense, 5’-GCAGGAGUUGGCUGAGAAG-3’, antisense, 5’ CUUCUCAGCCAACUCCUGC-3’).

### Stable cell line establishment

CRISPR-Cas9-mediated XRCC4 knockout was achieved in HCT116, MCF7, and A549 cell lines as previously described [35]. A synthesized single-guide RNA (sgRNA) for XRCC4 (XRCC4_gRNA_FW: CACCGTTACTGATGGTCA TTCAGCA, XRCC4_gRNA_RV: AAACTGCTGAATGACCATCAGTAAC) was inserted into the pSpCas9 (BB)-2A-Puro (PX459) V2.0 vector (Addgene, #62988, USA). HCT116, MCF7, and A549 cell lines were transfected with the sgRNA/Cas9 plasmid using Lipofectamine 2000 (Invitrogen, #11668027, USA) according to the manufacturer’s protocol, and subsequent selection of cells was carried out using puromycin. XRCC4-WT and XRCC4-T308A DNAs were cloned into the pMSCV-hygro/FLAG vector. The cloned retroviral constructs were transfected with the packaging plasmids pCMV-VSV-G and pCMV-Gag-Pol into HEK293T cells using Lipofectamine 2000. The retroviral supernatants were used to infect the previously generated XRCC4 knockout HCT116, MCF7, and A549 cells in the presence of 4 μg/mL of polybrene. The virally infected cells were selected with hygromycin. The XRCC4 knockout and reconstitution of XRCC4 were confirmed by western blotting.

### Western blotting and immunoprecipitation

Cells were lysed with RIPA buffer (50 mM Tris-HCl, pH 7.4, 150 mM NaCl, 2 mM EDTA, 1% NP-40, 0.1% SDS, and 0.5% sodium deoxycholate) or NP-40 lysis buffer (50 mM Tris-HCl, pH 7.4, 150 mM NaCl, 2 mM EDTA, and 1% NP-40) for immunoprecipitation and western blotting, supplemented with a EDTA-free protease inhibitor cocktail (Roche, Mannheim, Germany). For immunoprecipitation (IP) of exogenous FLAG-XRCC4, cell lysates underwent incubation with agarose-conjugated anti-FLAG antibody (MBL, Woburn, USA) for 2 h at room temperature (RT) or overnight at 4 °C. For immunoprecipitation of endogenous XRCC4, cell lysates were incubated with anti-XRCC4 antibody (Santa Cruz, #sc-271087, USA) and with agarose-conjugated protein A/G (Santa Cruz, #sc-2003, USA). Normal mouse IgG (Santa Cruz, #sc-2025, USA) antibody was used for control. The immunoprecipitated proteins were washed with the wash buffer (150 mM NaCl, 2 mM EGTA, 2 mM MgCl_2_, 20 mM HEPES, pH 7.4, and 0.1% NP-40) and went through elution with 2X sodium dodecyl sulfate (SDS) loading buffer. For western blotting, the samples were subjected to SDS-PAGE, transferred to nitrocellulose membrane, blocked in TBST + 5% skim milk, and incubated with the relevant specific antibodies. Following antibody incubation, signal detection utilized WestGlow chemiluminescent substrate (Biomax, #BWE0200, Korea) and FUJI RX-N X-ray film (FUJI, Japan). Signal quantification was conducted using ImageJ software (National Institutes of Health, https://imagej.nih.gov/ij/). The antibodies used for Western blotting or IP are listed in Table S1.

### In vivo ubiquitination assay

The ubiquitination of XRCC4 was analyzed following a previously established protocol [36]. In brief, cells were lysed in RIPA buffer, and SDS was added to the supernatants to a final concentration of 2%, followed by boiling for 10 min to denature the proteins. The samples were then diluted and rotated at 4 °C for 30 min. After centrifugation at 13,000 rpm for 20 min, the supernatants were subjected to immunoprecipitation with an XRCC4 antibody (Santa Cruz Biotechnology) overnight at 4 °C. Protein A/G was subsequently added, and the samples were rotated for an additional 2 h at 4 °C. The immunoprecipitated XRCC4 was washed five times with RIPA buffer before elution. Western blotting was then performed, and ubiquitination was detected using a K48-linked ubiquitin-specific antibody (Cell Signalling Technology, Danvers, MA, USA).

### Protein isolation and digestion

The eluate of XRCC4 was subjected to electrophoresis using Bolt 4–12% Bis-Tris Plus Gels (Thermo Fisher Scientific). Protein bands were excised, destained, and washed. Subsequently, proteins underwent a reduction using 20 mM DTT and alkylation using 55 mM iodoacetamide (IAA). Following dehydration, proteins were digested overnight at 37 °C with a Trypsin/LysC mix (12.5 ng/μL; Promega) in 50 mM ammonium bicarbonate. Peptides were then serially extracted from the gel using 10% formic acid (FA), 50% (v/v) acetonitrile (ACN) in 0.1% FA, and 80% ACN in 0.1% FA. The extracted peptides were dried and stored at −20 °C.

### Mass spectrometry analysis

For the identification of *O*-GlcNAc sites, the extracted peptides were diluted in 0.1% FA (Solvent A). LC-MS/MS analysis was performed in triplicate using an Easy nanoLC 1200 system integrated with an Orbitrap Fusion Lumos Tribrid mass spectrometer (Thermo Fisher Scientific, San Jose, CA). Peptides were initially loaded onto a C18 trap column (Acclaim PepMap100, Thermo Fisher Scientific, 75 μm × 2 cm, 100 Å) and then separated on a C18 analytical column (PepMap RSLC, Thermo Fisher Scientific, 75 μm × 50 cm, 100 Å) with a 70 minutes linear gradient from 5 to 38% solvent B (0.1% FA in ACN) with a flow rate of 300 nL/min. The column spray voltage was calibrated to 1.9 kV, and the heated capillary was maintained at 275 °C. The Q-Exactive operated in data-dependent acquisition mode, performing an MS survey scan followed by ten MS/MS scans of the most prevalent ions. The full MS scan range was 400 to 1400 m/z, with a dynamic exclusion period of 30 s. Upon detection of oxonium product ions (m/z 204.0867, 138.0545) in the higher-energy collisional dissociation (HCD) spectra, EThcD with a user-defined charge-dependent reaction time and 15 or 17% HCD supplemental activation was performed on the same precursor ion. The EThcD-MS/MS scans were acquired at a resolution of 30,000, with the AGC target adjusted to 1e5 and a maximum injection time of 120 ms. For the XRCC4 interactome analysis, peptides were similarly resuspended in 0.1% FA (Solvent A) and probed via LC-MS/MS using a Dionex 3000 UHPLC system integrated with a Q-Exactive™ mass spectrometer (Thermo Fisher Scientific, Germany) in triplicate. Peptides were loaded onto a C18 trap column (Acclaim PepMap100, Thermo Fisher Scientific, 75 μm × 2 cm, 100 Å) and separated on a C18 analytical column (PepMap RSLC, Thermo Fisher Scientific, 75 μm × 50 cm, 100 Å) using a 65 minutes linear gradient from 5 to 35% solvent B (0.1% FA in ACN) with a flow rate of 300 nL/min. The column spray voltage was calibrated to 2.2 kV, and the heated capillary was maintained at 250 °C. The normalized collision energy was set at 27 eV. The Q-Exactive operated in data-dependent acquisition mode, with an MS survey scan followed by ten MS/MS scans of the most prevalent ions. The total MS scan range was 400 to 1400 m/z, with a dynamic exclusion period of 20 s.

### Quantitative RT-PCR analysis

TRIzol (Invitrogen) was used for total RNA extraction, and reverse transcription (RT) for cDNA synthesis was carried out using ReverTra Ace qPCR RT Master Mix (Toyobo, #FSQ-201, Japan). Quantitative RT-PCR utilized TB Green Premix Ex Taq II (Takara, #RR820, Japan), and detection was carried out using the CFX Duet real-time PCR system (Bio-Rad) according to the manufacturer’s instructions. The oligonucleotide sequences are as follows: β-actin_For AGA GCT ACG AGC TGC CTG AC, β-actin_Rev AGC ACT GTG TTG GCG TAC AG, XRCC4_For TGG ACT GGG ACA GTT TCT GA, XRCC4_Rev TCA GTTCACCAACATATTTCCC.

### NHEJ and HR reporter plasmid assay

HeLa cell lines, stably integrated copy of the artificial recombination substrate DR-GFP with an I-SceI site (HR) or EJ5-GFP with two I-SceI sites (NHEJ), were transfected with I-Sce1 (Addgene, #26477) using Lipofectamine 2000 (Invitrogen) and incubated with 10 μg/mL bleomycin for 48 h. GFP expression was obtained by Fluorescence Activated Cell Sorting (FACS).

### Cell viability, transwell invasion, and soft agar colony formation assays

To measure cell viability, 1 × 10^3^– 1 × 10^4^ cells/well were plated in 96-well plates. At each time point following cell attachment, 10 μL of Quanti-Max WST-8 cell viability assay solution (Biomax, #QM1000, Korea) was added to each well, and the cells were maintained in incubation for 1 h at 37 °C. The absorbance was measured using a Multiskan Sky microplate spectrophotometer (Thermo Fisher Scientific) at 450 nm.

Invasion assay was conducted in a 24 mm Transwell with a 0.8-μm insert utilizing Corning Matrigel Matrix (Corning, #354234, USA) according to the manufacturer’s instructions. The Matrigel-coated Transwell insert upper chamber was seeded with 2.5 × 10^4^ cells/well and was incubated for 4 days at 37 °C with 5% CO_2_. Invading cells were quantified using the Quanti-Max WST-8 cell viability assay solution (Biomax, #QM1000, Korea).

For the soft agar colony formation assay, the CytoSelect 96-well Cell Transformation Assay (Cell Biolabs, USA) was employed according to the manufacturer’s instructions. Briefly, 2 × 10^5^ cells/well were seeded in soft agar. After incubation for 1 and 14 days, the soft agar was solubilized, and cells were lysed using lysis buffer. The resulting cell lysates were incubated with the CyQUANT GR Dye, and the readings were taken at the 485/520 nm filter set.

### Animal experiment

For azoxymethane/dextran sodium sulfate (AOM/DSS)-induced colon cancer, C57BL/6 J mice were purchased from DBL (DBL Co., Ltd., Eumseong, South Korea). The Institutional Animal Care and Use Committees of the Laboratory Animal Research Center at Yonsei University approved the experiments (IACUC-A-20221018-41). Twelve male C57BL/6J mice were selected to generate the AOM/DSS model. Ten mice were injected intraperitoneally with AOM (10 mg/kg). A week later, 1.5% DSS solution was prepared in drinking water for 7 days, and then the mice were provided regular sterile water for 14 weeks. All the water intake was not limited. Another two mice in the control group received intraperitoneal injection with an equal volume of phosphate-buffered saline. Animals were euthanized by CO_2_ asphyxiation at 16 weeks after the injection.

For the Xenograft experiment, BALB/c nude mice were purchased from DBL (DBL Co., Ltd., Eumseong, South Korea). The Institutional Animal Care and Use Committees of the Laboratory Animal Research Center at Yonsei University approved the experiments (IACUC-A-20221018-41). XRCC4 knockout, XRCC4 WT-expressing stable cells, and XRCC4 T308A-expressing stable cells were harvested using trypsin solution and washed with PBS. The cells were then suspended at a concentration of 1 × 10^7^ per 100 μL of PBS and injected into 5-week-old male BALB/c nude mice obtained from DBL (*n* = 6 per group). Tumor size and weight were assessed 2 months post-injection.

### Statistical analysis

All quantitative data were expressed as the mean ± SD from at least three independent experiments. Statistical analyses were performed using a two-tailed Student’s *t*-test for two groups and one-way analysis of variance (ANOVA) for multiple groups. Statistical significance was set at *P* < 0.05. Statistical significance is denoted as *P < 0.05, **P < 0.01, and ***P < 0.001

## Results

### Cellular *O*-GlcNAcylation levels modulate XRCC4 protein levels in cancers

Cancer cells exhibit elevated *O*-GlcNAc levels compared to normal cells, which is considered a hallmark of cancer [24]. Additionally, XRCC4 expression is also upregulated in cancer cells [33]. However, the link between XRCC4 and *O*-GlcNAcylation in cancer remains unclear. To address this, we first investigated the relationship between *O*-GlcNAcylation, XRCC4 expression, and cancer.

**Fig. 1:**
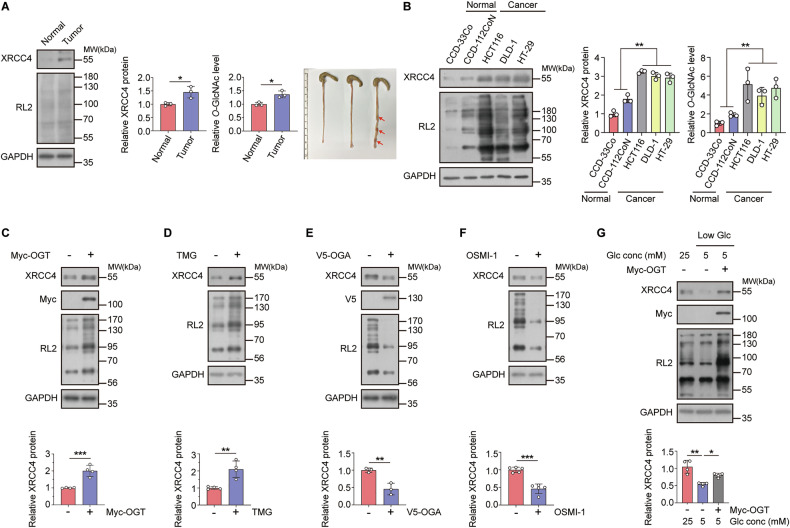
XRCC4 protein levels are regulated by cellular *O*-GlcNAcylation levels in colon cancer tissues and cell lines. **A** Colon tumors were induced in mice using azoxymethane (AOM) and dextran sulfate sodium (DSS). The levels of *O*-GlcNAcylated proteins were assessed by RL2 blotting. The levels of cellular *O*-GlcNAcylation and XRCC4 protein were compared between normal colon tissue and colon tumor (red arrow), normalizing the expression to GAPDH levels (*n* = 3). **B** Cellular *O*-GlcNAcylation and XRCC4 protein levels were compared between colon normal and cancer cell lines. XRCC4 expression was normalized to GAPDH levels (*n* = 3). **C**–**F** Cellular *O*-GlcNAcylation levels were regulated by **C** OGT transfection (*n* = 4), **D** 2 μM Thiamet-G (TMG), an OGA inhibitor, treatment (*n* = 4), **E** OGA transfection (*n* = 3), or **F** 30 μM OSMI-1, an OGT inhibitor, treatment (*n* = 5). The cell lysates were subjected to immunoblotting with the indicated antibodies. XRCC4 expression was normalized to GAPDH levels. **G** HCT116 cells were cultured in media containing 5 or 25 mM glucose for 24 h and transfected with control vector or OGT for 24 h. XRCC4 expression was normalized to GAPDH levels (*n* = 4). **A**–**G** Data were represented as means ± SD. Statistical significance was determined by the two-tailed Student’s *t*-test or one-way ANOVA for multiple comparisons (*P < 0.05, **P < 0.01, and ***P < 0.001).

For this, we compared the XRCC4 protein and cellular *O*-GlcNAcylation levels between normal colon and colon tumor tissues using a mouse model of colitis-associated colon cancer [37]. We confirmed that XRCC4 and *O*-GlcNAc levels were higher in colon tumor tissues than in normal colon tissues (Fig. 1A). Similarly, elevated cellular *O*-GlcNAcylation and XRCC4 protein levels were observed in several colon cancer cell lines (HCT116, DLD1, and HT29) compared to non-cancer cell lines (CCD-33Co and CCD-112CoN) (Fig. 1B). These effects were also observed in breast and lung cancer cell lines (Fig. S1A, B). We examined the effects of global perturbations of *O*-GlcNAc levels on XRCC4 expression to identify the relationship between elevated cellular *O*-GlcNAc and XRCC4 protein levels in cancer cells. Increasing cellular *O*-GlcNAc levels through overexpression of OGT or treatment with thiamet-G (an OGA inhibitor) upregulated XRCC4 in HCT116 cells (Fig. 1C, D). In contrast, reducing cellular *O*-GlcNAc levels by OGA overexpression or OSMI-1 treatment (an OGT inhibitor) decreased XRCC4 levels (Fig. 1E, F). Similar effects were observed in breast cancer cell line MCF7 (Fig. S1C, D) and lung cancer cell line A549 (Fig. S1E, F).

Glucose serves as a crucial substrate for *O*-GlcNAc modification, with high glucose levels often leading to DNA damage through increased reactive oxygen species (ROS) production [20, 38, 39]. Therefore, we investigated whether extracellular glucose concentrations affect XRCC4 levels. XRCC4 expression was higher under 25 mM glucose conditions than under 5 mM glucose conditions. OGT overexpression in HCT116 cells and other cancer cell lines reversed the decrease in XRCC4 levels under low-glucose conditions (Fig. 1G and Fig. S1G, H). These findings indicate a positive correlation between cellular *O*-GlcNAcylation levels and XRCC4 expression in cancer cells.

### Cellular *O*-GlcNAcylation levels regulate XRCC4 protein stability by affecting its ubiquitin-dependent proteasomal degradation

**Fig. 2:**
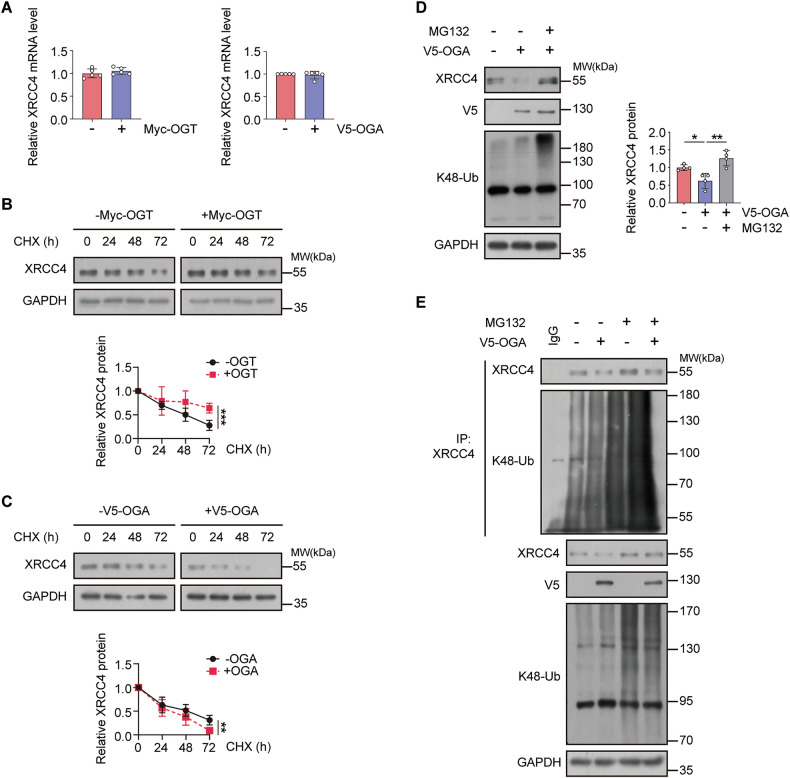
XRCC4 protein levels decrease in response to cellular *O*-GlcNAcylation levels in a proteasome-dependent manner. **A** Total RNA was extracted from HCT116 cells transfected with OGT or OGA for 24 h. XRCC4 mRNA levels were quantified by RT-qPCR and normalized to β-actin levels (*n* = 5). **B**, **C** HCT116 cells were transfected with **B** OGT (*n* = 5) or **C** OGA (*n* = 5) for 24 h, followed by 200 μg/mL cycloheximide (CHX), a protein synthesis inhibitor, treatment. Cells were collected at the indicated times. XRCC4 degradation rates were assessed by immunoblotting. XRCC4 expression was normalized to GAPDH levels. **D**, **E** HCT116 cells were transfected with OGA for 24 h and treated with 20 μM MG132, a proteasome inhibitor, for 6 h. **D** The cell lysates were subjected to immunoblotting with the indicated antibodies. XRCC4 expression was normalized to GAPDH levels (*n* = 4). **E** For the ubiquitination assay, the cell lysates were immunoprecipitated with the XRCC4 antibody. The polyubiquitination levels of XRCC4 were analyzed by immunoblotting with K48-Ub antibody. **A**–**D** Data were represented as means ± SD. Statistical significance was determined by the two-tailed Student’s *t*-test or one-way ANOVA for multiple comparisons (*P < 0.05, **P < 0.01, and ***P < 0.001).

We investigated the mechanism underlying the positive association between cellular *O*-GlcNAcylation and XRCC4 levels by considering two potential explanations: increased production of XRCC4 or inhibition of XRCC4 degradation. We analyzed how altering global *O*-GlcNAc levels through overexpression of OGT or OGA affected XRCC4 mRNA levels in the HCT116 cell line. Interestingly, changes in the cellular *O*-GlcNAc levels did not substantially impact XRCC4 mRNA levels (Fig. 2A). Similar results were observed in MCF7 and A549 cell lines (Fig. S2A, B). These findings suggest that *O*-GlcNAcylation predominantly influences XRCC4 protein stability rather than its mRNA levels. To further investigate this, we assessed XRCC4 protein half-life following cycloheximide (CHX) treatment to block protein synthesis. Elevated *O*-GlcNAcylation extended the half-life of the XRCC4 protein, whereas reduced *O*-GlcNAcylation had the opposite effect in HCT116, MCF7, and A549 cell lines (Fig. 2B, C and Fig. S2C–F). The ubiquitin–proteasome system plays a crucial role in protein turnover [40]. We investigated how cellular *O*-GlcNAc levels influence this pathway. OGA overexpression reduced endogenous XRCC4 protein levels, which were restored in the presence of the proteasome inhibitor MG132 (Fig. 2D). Moreover, downregulation of *O*-GlcNAc levels considerably increased K48-linked ubiquitination of XRCC4 (Fig. 2E). These results suggest that cellular *O*-GlcNAcylation modulates XRCC4 protein levels by inhibiting ubiquitin-dependent proteasomal degradation.

### TRIM21-mediated ubiquitin-dependent proteasomal degradation of XRCC4 is modulated by cellular *O*-GlcNAc levels

**Fig. 3:**
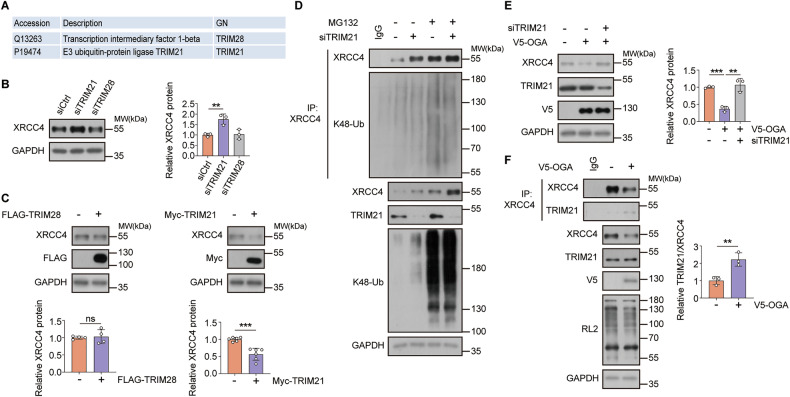
TRIM21 is the potential E3 ligase for XRCC4 in response to cellular *O*-GlcNAcylation levels. **A** Table showing the candidates of E3 ubiquitin ligases for XRCC4 as predicted by analyzing the interactome of XRCC4. **B** HCT116 cells were transfected with TRIM28 or TRIM21 siRNAs for 48 h. The cell lysates were immunoblotted with the indicated antibodies. XRCC4 expression was normalized to GAPDH levels (*n* = 3). **C** HCT116 cells were transfected with FLAG-TRIM28 (*n* = 4) or Myc-TRIM21 (*n* = 6) for 24 h. The cell lysates were immunoblotted with the indicated antibodies. XRCC4 expression was normalized to GAPDH levels. **D** HCT116 cells were transfected with TRIM21 siRNA for 48 h and treated with 20 μM MG132 for 6 h. For the ubiquitination assay, the cell lysates were immunoprecipitated with the indicated antibodies. The polyubiquitination levels of XRCC4 were analyzed by immunoblotting with K48-Ub antibody. **E** HCT116 cells were transfected with TRIM21 siRNA for 48 h and with OGA for 24 h. The cell lysates were subjected to immunoblotting with the indicated antibodies. XRCC4 expression was normalized to GAPDH levels (*n* = 3). **F** HCT116 cells were transfected with OGA for 24 h. For co-immunoprecipitation, the cell lysates were immunoprecipitated with the XRCC4 antibody. The affinity levels between XRCC4 and TRIM21 were analyzed by immunoblotting with the TRIM21 antibody. Co-immunoprecipitated TRIM21 levels were normalized to immunoprecipitated XRCC4 levels (*n* = 3). **B**, **C**, **E**, **F** Data were represented as means ± SD. Statistical significance was determined by the two-tailed Student’s *t*-test or one-way ANOVA for multiple comparisons (**P < 0.01, ***P < 0.001, ns not significant).

Cellular *O*-GlcNAcylation influences the ubiquitin-dependent proteasomal degradation of XRCC4; however, the precise mechanism remains unclear, as the specific E3 ligases responsible for this degradation have not been identified. We identified E3 ligase candidates responsible for XRCC4 degradation via interactome analysis of the XRCC4 protein (Table S2). We found that TRIM28 and TRIM21 interacted with XRCC4 in HCT116 cells (Fig. 3A). We knocked down each candidate protein in HCT116 cells using siRNA to determine the primary E3 ligase of XRCC4. TRIM21 downregulation increased XRCC4 levels, whereas no significant difference was observed with TRIM28 knockdown (Fig. 3B). This was consistent across MCF7 and A549 cell lines (Fig. S3A, B). Moreover, we assessed the effects of FLAG-TRIM28 and Myc-TRIM21 overexpression on endogenous XRCC4 levels. Myc-TRIM21 transfection decreased the XRCC4 levels, whereas FLAG-TRIM28 overexpression had no statistically significant impact (Fig. 3C). We treated HCT116 cells with siRNA targeting TRIM21 and conducted an in vivo ubiquitination assay to confirm the direct effect of TRIM21 on the ubiquitination status of XRCC4. siTRIM21 reduced K48-linked XRCC4 ubiquitination, suggesting that TRIM21 is a substantial E3 ligase candidate for XRCC4 (Fig. 3D).

We decreased cellular *O*-GlcNAc levels by OGA overexpression while knocking down TRIM21 to determine whether TRIM21 mediates XRCC4 degradation under conditions of altered *O*-GlcNAc levels. Consistent with our previous findings, XRCC4 stability decreased after V5-OGA overexpression. However, siTRIM21 restored XRCC4 protein levels (Fig. 3E). Two possible explanations exist for how cellular *O*-GlcNAc levels affect XRCC4 ubiquitination in a TRIM21-dependent manner, either by reducing TRIM21 levels or hindering the interaction between TRIM21 and XRCC4. TRIM21 levels remained unchanged despite changes in *O*-GlcNAc levels (Fig. S3C, D). Subsequently, we investigated whether *O*-GlcNAc levels influenced the interaction between TRIM21 and XRCC4. Co-immunoprecipitation assays using HCT116 cells demonstrated that decreased cellular *O*-GlcNAc levels increased the interaction between XRCC4 and TRIM21 (Fig. 3F). Thus, *O*-GlcNAc modification inhibits the ubiquitin-dependent XRCC4 degradation by reducing the interaction between XRCC4 and its E3 ubiquitin ligase, TRIM21.

### Thr308 is the major *O*-GlcNAcylation site of XRCC4

**Fig. 4:**
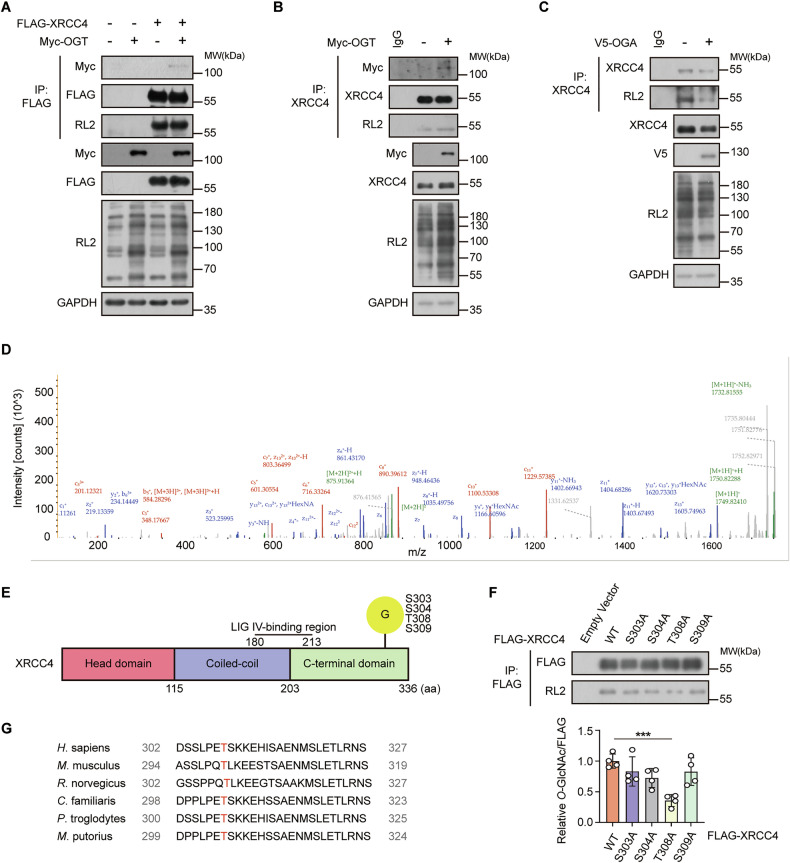
XRCC4 is *O*-GlcNAcylated on Thr308. **A** HCT116 cells were transfected with FLAG-XRCC4 or Myc-OGT for 24 h. The cell lysates were immunoprecipitated using FLAG beads. Exogenous *O*-GlcNAcylation of XRCC4 was detected by immunoblotting with RL2 antibody. For co-immunoprecipitation, the immunoprecipitated lysates were immunoblotted with Myc antibody. **B**, **C** HCT116 cells were transfected with **B** Myc-OGT or **C** V5-OGA for 24 h. The cell lysates were immunoprecipitated using the XRCC4 antibody. Endogenous *O*-GlcNAcylation of XRCC4 was detected by immunoblotting with RL2 antibody. **B** For co-immunoprecipitation, the immunoprecipitated lysates were immunoblotted with Myc antibody. **D** Putative *O*-GlcNAc sites of XRCC4 were identified by EThcD-MS/MS. *O*-GlcNAcylated XRCC4 peptides, 297-ENSRPDSSLPEtSK-310, is shown. **E** A schematic drawing of the putative XRCC4 *O*-GlcNAcylation sites, including Ser303, Ser304, Thr308, and Ser309, based on mass spectrometry data. **F** HCT116 cells were transiently transfected with each indicated FLAG-XRCC4 point mutant. The cell lysates were immunoprecipitated using FLAG beads. *O*-GlcNAcylation of each FLAG-XRCC4 point mutant was detected by immunoblotting with RL2 antibody. *O*-GlcNAcylated FLAG-XRCC4 was normalized to immunoprecipitated FLAG-XRCC4 (*n* = 4). Data were represented as means ± SD. Statistical significance was determined by the two-tailed Student’s *t*-test (***P < 0.001). **G** Cross-species sequence alignment of XRCC4.

OGT catalyzes *O*-GlcNAcylation of nuclear and cytoplasmic proteins [28]. Our previous interactome study showed an interaction between XRCC4 and OGT, implying that XRCC4 undergoes *O*-GlcNAc modifications in HCT116 colon cancer cells [14]. Thus, *O*-GlcNAc modification of XRCC4 may affect its interaction with TRIM21. We verified this by transfecting FLAG-XRCC4 and Myc-OGT to HCT116 cells and observed that OGT *O*-GlcNAcylated the exogenous XRCC4 (Fig. 4A). Furthermore, HCT116 cell lysates were immunoprecipitated with an XRCC4 antibody and detected using an RL2 antibody. The *O*-GlcNAcylation level of endogenous XRCC4 was elevated upon OGT overexpression and reduced following OGA overexpression (Fig. 4B, C). Conversely, knockdown of OGT led to a decrease in XRCC4 *O*-GlcNAcylation, whereas knockdown of OGA resulted in its increase (Fig. S4A, B). Co-immunoprecipitation further confirmed the interaction of XRCC4 with both exogenous OGT (Fig. 4A, B) and endogenous OGT (Fig. S4A).

Given the confirmed interaction between XRCC4 and OGT, as well as the observed *O*-GlcNAcylation of XRCC4, we sought to identify its major *O*-GlcNAcylation site using mass spectrometry (MS). For MS analysis, HEK293 cells were used instead of HCT116 cells to obtain sufficient FLAG-XRCC4 (Fig. S4D). We transiently overexpressed FLAG-XRCC4 and Myc-OGT in HEK293 cells to confirm XRCC4 *O*-GlcNAcylation (Fig. S4C). MS analysis identified four *O*-GlcNAcylation sites: Serine 303 (Ser303; S303), Serine 304 (Ser304; S304), Threonine 308 (Thr308; T308), and Serine 309 (Ser309; S309) (Fig. 4D, E and Fig. S4E). Based on this, we constructed *O*-GlcNAc-deficient point mutants at each *O*-GlcNAc site in XRCC4 by substituting serine (Ser) or threonine (Thr) with alanine (Ala). Among these mutants, substituting Thr308 with Ala (FLAG-XRCC4 T308A) demonstrated the most pronounced effect on the *O*-GlcNAc levels compared to the FLAG-XRCC4 wild-type (WT) (Fig. 4F), suggesting that this site is the major *O*-GlcNAcylation site on XRCC4. Sequence alignment showed high conservation of Thr308 in XRCC4 across species (Fig. 4G). These results indicated that XRCC4 is *O*-GlcNAcylated, with Thr308 being the major *O*-GlcNAcylation site.

### *O*-GlcNAcylation of XRCC4 at Thr308 is critical for XRCC4 stability

**Fig. 5:**
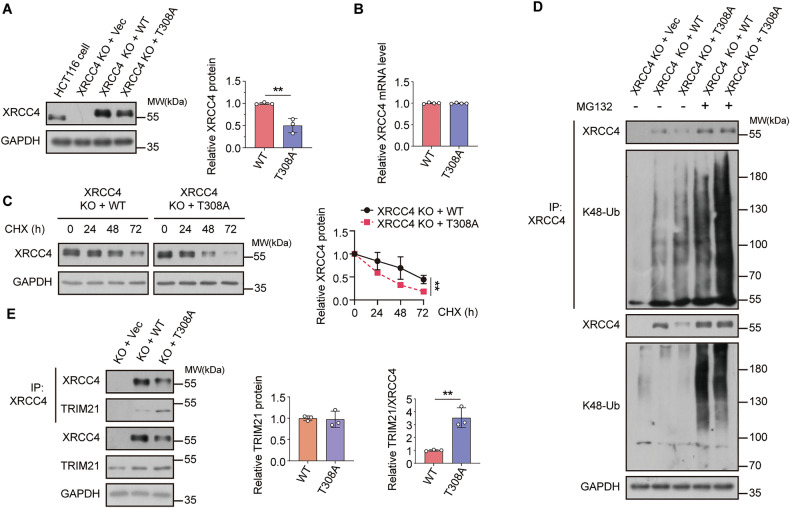
*O*-GlcNAc modification of XRCC4 at Thr308 affects cancer cells by blocking its interaction with TRIM21. **A** FLAG-XRCC4 WT or T308A were stably expressed in HCT116 XRCC4 KO cells. The cell lysates were immunoblotted with the indicated antibodies. XRCC4 expression was normalized to GAPDH levels (*n* = 3). **B** Total RNA was extracted from HCT116 XRCC4 KO cells stably expressing WT or T308A XRCC4. XRCC4 mRNA levels were quantified by RT-qPCR and normalized to β-actin levels (*n* = 4). **C** HCT116 XRCC4 KO cells stably expressing WT or T308A XRCC4 were treated with 200 μg/mL cycloheximide (CHX). Cells were collected every 24 h and the cell lysates were immunoblotted with the indicated antibodies. XRCC4 expression was normalized to GAPDH levels (*n* = 4). **D** HCT116 XRCC4 KO cells stably expressing WT or T308A XRCC4 were treated with 20 μM MG132 for 6 h. For the ubiquitination assay, the cell lysates were immunoprecipitated using the XRCC4 antibody. The polyubiquitination levels of XRCC4 were analyzed by immunoblotting with K48-Ub antibody. **E** For co-immunoprecipitation, the cell lysates from HCT116 XRCC4 KO cells stably expressing WT or T308A XRCC4 were subjected to immunoprecipitation with XRCC4 antibody. The affinity levels between XRCC4 and TRIM21 were analyzed by immunoblotting with the TRIM21 antibody. TRIM21 expression was normalized to GAPDH levels (*n* = 3). Co-immunoprecipitated TRIM21 levels were normalized to immunoprecipitated XRCC4 levels (*n* = 3). **A**–**C**, **E** Data were represented as means ± SD. Statistical significance was determined by the two-tailed Student’s *t*-test (**P < 0.01).

We generated an XRCC4-knockout (XRCC4 KO) HCT116 cell line using the CRISPR-Cas9 system to evaluate whether Thr308 *O*-GlcNAcylation is responsible for XRCC4 degradation. We established stable cell lines from the XRCC4-KO HCT116 cell line expressing either FLAG-XRCC4 WT or FLAG-XRCC4 T308A via viral infection. We observed that both the *O*-GlcNAcylation of XRCC4 and XRCC4 expression decreased in the cell line stably expressing the XRCC4 T308A mutant compared to XRCC4 WT (Fig. S5A and Fig. 5A). This finding was confirmed in MCF7 and A549 cell lines expressing FLAG-XRCC4 WT or FLAG-XRCC4 T308A mutant (Fig. S5B, C).

Consistent with the results presented in Fig. 2A–C, we compared XRCC4 mRNA levels and protein half-life between FLAG-XRCC4 WT and FLAG-XRCC4 T308A mutant cell lines. The difference in mRNA levels was insignificant; however, FLAG-XRCC4 WT exhibited a longer half-life than the FLAG-XRCC4 T308A mutant (Fig. 5B, C). Additionally, the K48-linked polyubiquitination of the FLAG-XRCC4 T308A mutant was higher than that of the FLAG-XRCC4 WT, and this difference was considerably increased in the presence of MG132, consistent with our previous findings in Fig. 2 (Fig. 5D). These results highlight the critical role of *O*-GlcNAcylation of XRCC4 at Thr308 in enhancing XRCC4 stability. Furthermore, we investigated the interactions between TRIM21 and either FLAG-XRCC4 WT or FLAG-XRCC4 T308A. As anticipated, the interaction between FLAG-XRCC4 WT and TRIM21 was weaker than that between FLAG-XRCC4 T308A and TRIM21 (Fig. 5E). These findings suggest that *O*-GlcNAcylation of XRCC4 at Thr308 protects XRCC4 from ubiquitin-dependent degradation by reducing its affinity for the E3 ligase TRIM21. Consistent with this mechanism, overexpression of OGT and OGA induced a more pronounced increase or decrease in XRCC4 levels, respectively, in FLAG-XRCC4 WT cells than in FLAG-XRCC4 T308A cells, further underscoring the importance of Thr308 *O*-GlcNAcylation on regulating XRCC4 levels (Fig. S5D, E).

### *O*-GlcNAcylation of XRCC4 at Thr308 modulates XRCC4-dependent resistance of cancer cells to DNA double-strand break damage

**Fig. 6:**
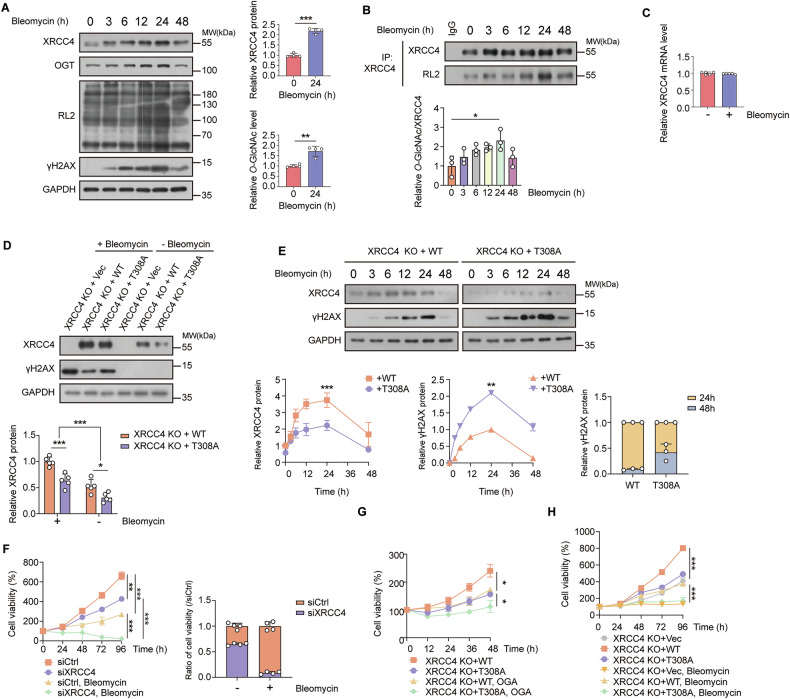
*O*-GlcNAcylation of XRCC4 at Thr308 protects cancer cells against cell death in response to DNA double-strand break damage. **A**, **B** HCT116 cells were treated with 10 μg/mL bleomycin. Cells were collected with the indicated time. **A** The cell lysates were immunoblotted with the indicated antibodies. XRCC4 and *O*-GlcNAcylation levels were normalized to GAPDH levels (*n* = 4). **B** Endogenous *O*-GlcNAcylation of XRCC4 at the indicated time was detected by immunoblotting with RL2 antibody. *O*-GlcNAcylated XRCC4 was normalized to immunoprecipitated XRCC4 (*n* = 3). **C** Total RNA was extracted from HCT116 cells treated with or without 10 μg/mL bleomycin for 24 h. XRCC4 mRNA levels were quantified by RT-qPCR and normalized to β-actin levels (*n* = 5). **D** HCT116 XRCC4 KO cells stably expressing WT or T308A XRCC4 were treated with or without 10 μg/mL bleomycin for 24 h. The cell lysates were immunoblotted with the indicated antibodies. XRCC4 expression was normalized to GAPDH levels (*n* = 5). **E** HCT116 XRCC4 knockout (KO) cells stably expressing either wild-type (WT) or T308A mutant XRCC4 were treated with 10 μg/mL bleomycin. Cells were harvested at the indicated time points, and whole-cell lysates were subjected to immunoblotting with the specified antibodies. XRCC4 and γH2AX expression levels were normalized to GAPDH. The bar graph depicts relative γH2AX levels at 24 and 48 h in HCT116 XRCC4 KO cells stably expressing WT or T308A XRCC4 (*n* = 3). **F** XRCC4 was knocked down using siRNA and treated with or without 10 μg/mL bleomycin. WST-8 assay was performed after treatment with bleomycin for the indicated time. Quantification of relative growth rates was performed at 96 h (*n* = 4). **G** HCT116 XRCC4 KO cells stably expressing WT or T308A XRCC4 were all treated with 10 μg/mL bleomycin and were transfected with either control vector or OGA. WST-8 assay was performed after treatment with bleomycin for the indicated time (*n* = 5). **H** HCT116 XRCC4 KO cells stably expressing WT or T308A XRCC4 were treated with or without 10 μg/mL bleomycin. WST-8 assay was performed after treatment with bleomycin for the indicated time (*n* = 4). **A**–**H** Data were represented as means ± SD. Statistical significance was determined by the two-tailed Student’s *t*-test (*P < 0.05, **P < 0.01, ***P < 0.001).

The NHEJ pathway is the primary mechanism for repairing IR-induced DSBs caused by exogenous and endogenous DNA-damaging factors in mammalian cells [8, 41]. Within this pathway, XRCC4 is a crucial protein that enables the interaction of DNA LIG4 with damaged DNA and controls LIG4 stability and activity [12, 42]. Regarding XRCC4’s functional significance, we examined the effect of XRCC4 *O*-GlcNAcylation on DNA damage response. DSBs were induced using bleomycin, a chemotherapeutic radiomimetic drug known to generate more homogeneous DSBs than IR [43]. Bleomycin treatment increased the γH2AX levels, a marker for DSBs, and they remained high for up to 24 h (Fig. 6A). The γH2AX levels subsequently decreased at 48 h, suggesting DSB resolution via DNA damage response, including the NHEJ pathway (Fig. 6A). We confirmed that both NHEJ and HR activities were elevated following 48 h of bleomycin exposure, suggesting that the enhancement of NHEJ contributes to the resolution of γH2AX levels in conjunction with HR (Fig. S6A). Additionally, we applied media change to remove bleomycin after a short period to mimic irradiation experiments, which induce acute initial DNA damage. γH2AX levels and XRCC4 levels continuously declined after media change (Fig. S6B).

OGT, XRCC4, and cellular *O*-GlcNAc levels closely correlated with the alterations in the γH2AX levels (Fig. 6A), and this pattern was consistent in MCF7 and A549 cell lines (Fig. S6C, D). Notably, XRCC4 *O*-GlcNAcylation peaked 24 h after bleomycin treatment (Fig. 6B). We conducted quantitative polymerase chain reaction (qPCR) analysis to measure the XRCC4 mRNA level with or without bleomycin treatment and found no significant difference (Fig. 6C). Thus, the bleomycin-induced increase in XRCC4 levels is attributable to enhanced protein stability rather than elevated XRCC4 mRNA expression. Accordingly, we hypothesized that upregulation of *O*-GlcNAcylation in response to DNA double-strand breaks promotes XRCC4 protein stability, thereby facilitating NHEJ pathway activation and contributing to efficient DSB resolution [44].

We validated this hypothesis by a series of experiments. First, we examined the effect of XRCC4 *O*-GlcNAcylation, especially Thr308 *O*-GlcNAcylation, on the XRCC4 expression and DNA damage. FLAG-XRCC4 T308A cells, which have lower XRCC4 *O*-GlcNAcylation levels than XRCC4 WT cells (Fig. S5A), also have lower XRCC4 protein levels and increased DNA damage (Fig. 6D). This trend persisted across a 48 h time course (Fig. 6E). Second, we examined whether differential XRCC4 protein levels contribute to variation in cellular resistance to bleomycin. Cells with XRCC4 knockdown exhibited a greater reduction in viability following bleomycin treatment compared to control cells (Fig. 6F). This trend was consistently observed in both MCF7 and A549 cell lines (Fig. S6E, F). Third, we aimed to establish a connection between (1) the impact of XRCC4 *O*-GlcNAcylation on XRCC4 protein levels and (2) its influence on bleomycin resistance. Reducing cellular *O*-GlcNAcylation levels by OGA overexpression led to a more pronounced decrease in bleomycin resistance in FLAG-XRCC4 WT cells than in FLAG-XRCC4 T308A cells (Fig. 6G). Given that FLAG-XRCC4 WT exhibits greater variability in *O*-GlcNAcylation levels than XRCC4 T308A, these findings suggest that XRCC4 *O*-GlcNAcylation— particularly at Thr308—plays a critical role in modulating bleomycin resistance. Fourth, we conducted cell growth assays with bleomycin treatment and found that XRCC4 WT-expressing cells exhibited markedly enhanced bleomycin resistance, whereas XRCC4 T308A-expressing cells exhibited bleomycin resistance similar to that of XRCC4-KO cells (Fig. 6H). These results collectively demonstrate the conclusion that *O*-GlcNAcylation of XRCC4 at Thr308 enhances its protein stability and promotes cellular resistance to DNA damage by facilitating efficient DNA repair.

### XRCC4 *O*-GlcNAcylation promotes cancer cell proliferation and tumor growth

**Fig. 7:**
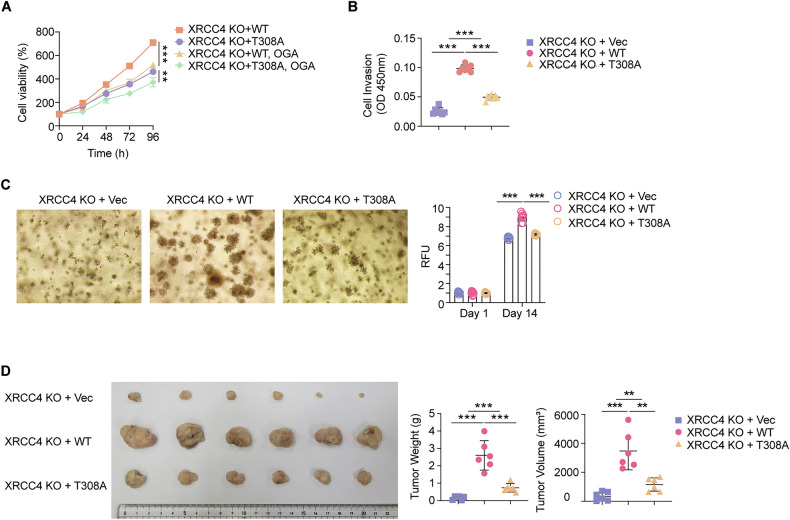
Thr308 *O*-GlcNAcylation of XRCC4 promotes cancer cell proliferation, invasion, colony formation, and tumor growth. **A** HCT116 XRCC4 KO cells stably expressing WT or T308A XRCC4 were transfected with OGA. WST-8 assay was performed after growth for the indicated time (*n* = 5). **B** HCT116 XRCC4 KO cells stably expressing WT or T308A XRCC4 were subject to a Transwell invasion assay. Invaded cells were measured by WST-8 assay (*n* = 7). **C** HCT116 XRCC4 KO cells stably expressing WT or T308A XRCC4 were subject to colony formation assays. Colony formation was quantified by using a 485/520 nm filter set (*n* = 8). **D** Each group of BALB/c nude mice (*n* = 6 per group) were injected with 1 × 10^7^ of the mentioned cells into the hypodermis. Tumor size and weight were quantified 60 days after injection. **A**–**D** Data were represented as means ± SD. Statistical significance was determined by the two-tailed Student’s *t*-test or one-way ANOVA for multiple comparisons (**P < 0.01, ***P < 0.001).

Previous studies have shown that XRCC4 participates in cell cycle regulation and functions as a pivotal tumor suppressor that plays a central role in controlling cancer progression [45]. We wanted to probe whether XRCC4 *O*-GlcNAcylation mediated the relationship between global *O*-GlcNAcylation levels and cancer cell growth. XRCC4 WT-expressing cells proliferated significantly more than those expressing XRCC4 T308A (Fig. 6H). Furthermore, reduction of global *O*-GlcNAcylation levels led to a more pronounced decrease in the cell proliferation of XRCC4 WT-expressing cells compared to XRCC4 T308A-expressing cells in HCT116, MCF7, and A549 cell lines (Fig. 7A and S7A, B). Given that XRCC4 WT exhibits greater variability in *O*-GlcNAcylation levels than XRCC4 T308A, these results indicate that XRCC4 *O*-GlcNAcylation mediates the relationship between global *O*-GlcNAc levels and cancer cell growth. Furthermore, cell invasion assays revealed a significant increase in invasion with FLAG-XRCC4 WT, whereas FLAG-XRCC4 T308A displayed only slightly higher invasion levels than those observed in HCT116 XRCC4 KO cells (Fig. 7B). To validate this effect over a longer time scale, we conducted soft agar colony formation assays. Cells expressing FLAG-XRCC4 WT produced larger colonies compared to XRCC4-KO cells. FLAG-XRCC4 T308A cells formed colonies that were similar in size to XRCC4-KO cells (Fig. 7C). Finally, we conducted tumor xenograft assays on BALB/c nude mice to confirm the role of *O*-GlcNAcylation on XRCC4 at Thr308 in tumor development (Fig. 7D). The size and weight of the tumors derived from XRCC4 WT-expressing cells were significantly larger and heavier than those from the other groups, whereas tumors from FLAG-XRCC4 T308A cells were slightly larger and heavier than those from HCT116 XRCC4-KO cells. Overall, *O*-GlcNAcylation of XRCC4 at Thr308 is critical for promoting cancer cell growth and tumorigenesis.

**Fig. 8:**
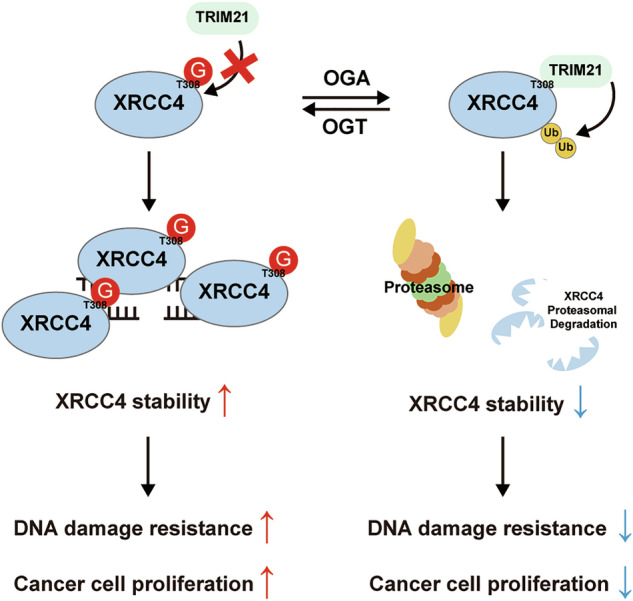
Schematic representation of the mechanism regulating XRCC4 stability through *O*-GlcNAcylation upon DNA damage. *O*-GlcNAcylation of XRCC4 at Thr308 increases the stability of the protein by inhibiting the interaction between XRCC4 and the E3 ligase TRIM21. Upregulated XRCC4 promotes resistance to DNA double-strand breaks and cancer cell proliferation.

Collectively, these findings demonstrate that *O*-GlcNAcylation of XRCC4 at Thr308 modulates the resistance of cancer cells to DNA DSB damage, dependent on XRCC4. Thus, *O*-GlcNAc levels regulate XRCC4 stability through the E3 ligase TRIM21, and *O*-GlcNAcylation of XRCC4 at Thr308 serves as a critical link between *O*-GlcNAc modification and XRCC4 function. This modification is particularly important for DNA damage response and cancer cell proliferation. XRCC4 Thr308 *O*-GlcNAcylation hinders TRIM21 and XRCC4 interaction. This prevents polyubiquitination of the protein, resulting in increased stability. Increased XRCC4 levels enabled the cancer cells to resist the stress of DNA damage (Fig. 8).

## Discussion

*O*-GlcNAcylation is upregulated in various cancer types and is linked to the metabolic reprogramming of tumor cells and the high-stress tumor microenvironment [46, 47]. Furthermore, different stress factors trigger the DDR or genomic instability, increasing the expression of DDR-related proteins such as XRCC4 and promoting cancer progression [48]. Despite evidence highlighting the critical role of *O*-GlcNAcylation-regulated DDR in tumor therapy, related research is limited and scattered. In the present study, we found a positive correlation between XRCC4 expression and cellular *O*-GlcNAcylation in multiple cancer types, especially in response to DSBs. Additionally, we explored cancer therapy strategies through DNA damage repair by modifying the *O*-GlcNAcylation of XRCC4 at Thr308.

We confirmed the correlation between intracellular *O*-GlcNAc and XRCC4 levels in cancer cells. XRCC4 and global *O*-GlcNAcylation levels were concurrently elevated in various cancer cell types. (Fig. 1A, B and Fig. S1A, B). Furthermore, a positive relationship between cellular *O*-GlcNAc and XRCC4 protein levels was observed when the expression levels of OGT and OGA were manipulated (Fig. 1C–F and Fig. S1C–F). Through interactome analysis, we identified TRIM21 as a previously unrecognized K48-dependent E3 ubiquitin ligase that regulates the proteasomal degradation of XRCC4 (Fig. 3A). Cellular *O*-GlcNAc levels influenced this degradation (Fig. 2B–E) due to altered interaction between XRCC4 and its E3 ligase TRIM21 (Fig. 3F).

We found that XRCC4 was *O*-GlcNAcylated (Fig. 4A–C and Fig. S4A–C). Using mass spectrometry, we identified four potential *O*-GlcNAcylation sites on XRCC4 (Fig. 4D, E and Fig. S4E). Experiments with point mutants identified Thr308 as the major *O*-GlcNAcylation site (Fig. 4F). However, given that the *O*-GlcNAcylation of XRCC4 was not entirely abolished when Thr308 was substituted with alanine (T308A), this suggests that the other three sites also influence XRCC4’s stability and function (Fig. 4F). Nonetheless, we confirmed that *O*-GlcNAcylation at Thr308 significantly impacts the stability of XRCC4 (Fig. 5A–C) when we generated stably XRCC4 T308A-expressing cell lines and replicated the previous experiments.

Previous studies have suggested that lower XRCC4 expression levels are associated with better results after chemotherapy or radiotherapy [49, 50]. Additionally, experimental studies indicate that the knockdown of XRCC4 or epigenetic inhibition of LIG4/XRCC4/XLF complex formation increases cancer cell sensitivity to anticancer agents [51]. Therefore, we investigated how *O*-GlcNAcylation of XRCC4 affects cancer cell responses to DNA DSBs induced by bleomycin, a radiomimetic and chemotherapeutic drug commonly used in DNA repair studies and a DNA damage-inducing agent [43, 52, 53]. Compared with other DSB-inducing factors, bleomycin generates more homogeneous DSBs than ionizing radiation (IR) [43]. Additionally, DSBs induced by IR and chemotherapeutic agents, including bleomycin, more closely mimic naturally occurring DSBs than those induced by restriction enzymes [54]. XRCC4 is particularly sensitive to bleomycin compared to other proteins in the NHEJ pathway, as shown by a substantial decrease in cancer cell survival rates in XRCC4 knockout cells [43]. We found that bleomycin-induced DNA damage upregulated cellular *O*-GlcNAc and XRCC4 levels in various cancer cell types (Fig. 6A and Fig. S6C, D). Furthermore, using the DNA damage marker γH2AX, we observed that DNA repair was less efficient in XRCC4 T308A-expressing cells compared to XRCC4 WT-expressing cells, resulting in increased cancer cell death (Fig. 6D–H). We also observed that DNA repair upon bleomycin treatment involves both the HR and NHEJ pathways (Fig. S6A). XRCC4 is primarily recognized as a key component of the NHEJ pathway [6, 9]. However, it can also influence the HR pathway indirectly through the regulation of HR-related proteins [55, 56] and by contributing to the choice between HR and NHEJ [57, 58]. Based on these considerations, we suggest that XRCC4 plays an important role in DNA repair following bleomycin-induced damage.

Bleomycin treatment led to an increase in XRCC4 protein levels even in XRCC4 T308A mutant cells, although the extent of this increase was lower compared to cells expressing XRCC4 WT. We hypothesize that this residual upregulation in the absence of the primary *O*-GlcNAcylation site at Thr308 may be attributed to several alternative mechanisms: (1) the presence of additional, minor *O*-GlcNAcylation sites on XRCC4; (2) bleomycin-induced modulation of other post-translational modifications such as phosphorylation [59] and SUMOylation [18], which may be implicated in the regulation of XRCC4 stability; (3) the influence of bleomycin on transcription factors known to bind the XRCC4 promoter, including androgen receptor (AR) [60], the JNK–cJUN complex, and ZNF281 [61]; and (4) the effect of bleomycin on non-coding RNA regulators of XRCC4 expression, such as miR-3065-5p and the long non-coding RNA ZFPM2-AS [62]. Nonetheless, we underscore that *O*-GlcNAcylation of XRCC4 at Thr308 constitutes the predominant mechanism by which bleomycin enhances XRCC4 protein levels. According to previous studies, XRCC4 is involved in cell cycle control, which critically affects cancer progression [45]. To investigate the specific effects of XRCC4 Thr308 *O*-GlcNAcylation, we conducted experiments without bleomycin treatment (Fig. 7). Physiological experiments showed that this *O*-GlcNAcylation site affected cell growth and tumorigenesis. These findings highlight the importance of *O*-GlcNAcylation at Thr308 for the function of XRCC4 in cancer growth and resilience.

Based on our findings, we proposed a model illustrating the crosstalk between *O*-GlcNAcylation and the DNA repair system in cancers (Fig. 8). *O*-GlcNAcylation of XRCC4 at Thr308 bridges the two systems. DNA damage signals trigger *O*-GlcNAcylation of XRCC4 at Thr308, hindering its interaction with the ubiquitin E3 ligase TRIM21. This enhances XRCC4 protein stability, increasing its levels and providing resilience to DNA damage in cancer cells. Similar mechanisms, where *O*-GlcNAcylation modulates protein stability and thereby acts as a switch, are observed in other pairs of proteins and E3 ligases like the Melanophilin/TRIM21 and SMAD4/GSK-3beta pairs [63, 64]. Importantly, this study is the first to identify *O*-GlcNAcylation of a core component within the LIG4/XLF/XRCC4 complex. Our previous research has shown that the NHEJ-related proteins XRCC4 and XRCC6 (Ku70) are part of the OGT interactome [14]. Additionally, XRCC6, DNA-PKcs, and XRCC5 (Ku80) have been identified as targets of *O*-GlcNAcylation [65, 66]. However, the core LIG4/XLF/XRCC4 complex, directly involved in DNA ligation, has not yet been examined for *O*-GlcNAcylation. We focused on XRCC4 because its stability and ability to bind to the DNA through LIG4 depend on it, making XRCC4 the hub of the LIG4/XLF/XRCC4 complex. Additionally, XRCC4-like factor protein (XLF) may be *O*-GlcNAcylated due to its structural similarity to XRCC4.

Our results suggest that downregulating XRCC4 Thr308 *O*-GlcNAcylation could be a promising target for combination therapy with radiotherapy or chemotherapy. Cancer cell resilience to DNA damage from therapy may rely on XRCC4, stabilized by *O*-GlcNAcylation at Thr308. Thus, reducing XRCC4’s *O*-GlcNAcylation level alongside conventional therapy may enhance cancer cell sensitivity to treatment. This approach can be applied using an OGA-fused nanobody that specifically targets XRCC4 to remove *O*-GlcNAcylation, thereby offering a novel cancer treatment strategy [67]. cJun, c-Fos, and Nup62 were successfully targeted for *O*-GlcNAc removal by OGA-fused nanobodies. Additionally, PROTACs linking XRCC4 and TRIM21 may facilitate XRCC4 ubiquitination despite its *O*-GlcNAc modification. We propose that targeting the *O*-GlcNAcylation of XRCC4 at Thr308 could be a promising strategy for cancer therapy.

## Supplementary information

Supplementary Data

Original Blot

## Data Availability

All data reported in this paper will be shared by the lead contact upon request. This paper does not report original code. Any additional information required to reanalyze the data reported in this paper is available from the lead contact upon request.
