# Supplementary material for: O-GlcNAcylation of XRCC4 controls its stability and confers resistance to DNA double-strand break damage in cancer cells
Source: Cell Death Dis. 2026 Jan 9;17(1):22. doi: 10.1038/s41419-025-08209-4 (PMC12789502; doi:10.1038/s41419-025-08209-4)
Supplement: Supplementary file 1 — Supplementary Data [file 41419_2025_8209_MOESM1_ESM.pdf]

Supplementary Information

O-GlcNAcylation on the DNA Repair Protein XRCC4 Controls its Stability and Confers Resistance to DNA Double-strand Break Damage in Cancer Cells

Jeong Yeon Ko[1], Tae Hyun Kweon[1,2], Hyeryeon Jung[3], Jingu Kang[1,2], Yeolhoe Kim[1], Yun Ju Kim[1], Donghyuk Shin[1], Seong Wook Yang[1], Myeong Min Lee[1], Jun Young Hong[1], Jae-Min Lim[4], Eugene C. Yi[3,5], Jin Won Cho[1,5], Won Ho Yang[*1,5]

[1]Department of Systems Biology, College of Life Science and Biotechnology, Yonsei University, 50 Yonsei-ro, Seodaemun-gu, Seoul 03722, Republic of Korea

[2]Interdisciplinary Program of Integrated OMICS for Biomedical Science, Graduate School, Yonsei University, 03722 Seoul. Republic of Korea

[3]Department of Molecular Medicine and Biopharmaceutical Sciences, School of Convergence Science and Technology and College of Medicine or College of Pharmacy, Seoul National University, 28 Yeongeon-dong, Jongno-gu, Seoul 03080, Republic of Korea

[4]Department of Chemistry, College of Natural Sciences, Changwon National University, Changwon 51140, Republic of Korea

[5]Glycosylation Network Research Center, Yonsei University, 50 Yonsei-ro, Seodaemun-gu, Seoul 03722, Republic of Korea

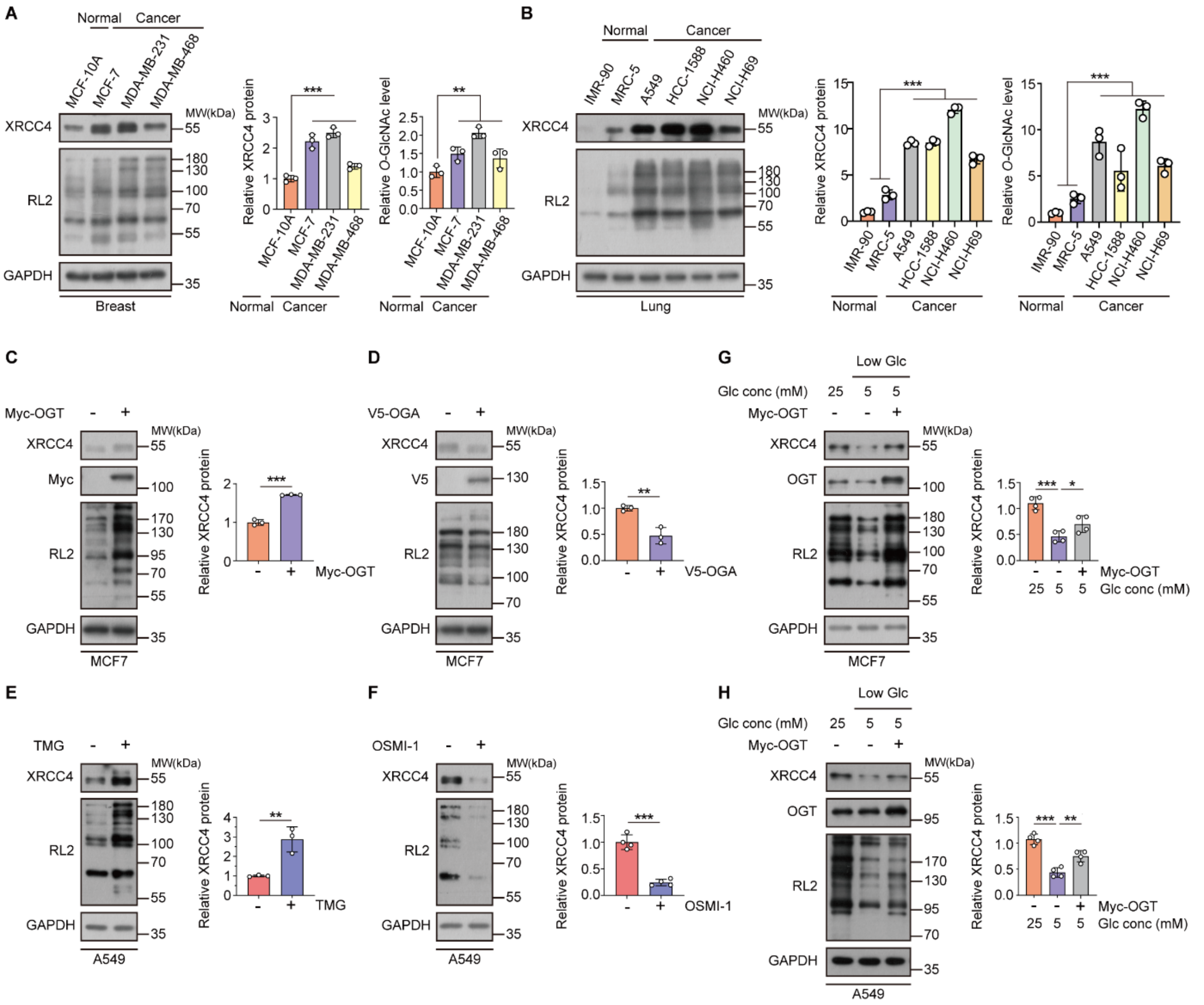

Figure S1. The stability of XRCC4 is regulated by cellular *O*-GlcNAcylation level in cancer cells. **A** Cellular *O*-GlcNAcylation and XRCC4 protein levels were compared between breast normal and cancer cell lines. XRCC4 expression was normalized to GAPDH levels (*n*=3). **B** Cellular *O*-GlcNAcylation and XRCC4 protein levels were compared between lung normal and cancer cell lines. XRCC4 expression was normalized to GAPDH levels (*n*=3). **C, D** Cellular *O*-GlcNAcylation levels were regulated by **(C)** OGT transfection (*n*=3) or **(D)** OGA transfection (*n*=3) in MCF7 cells. The cell lysates were immunoblotted with the indicated antibodies. XRCC4 expression was normalized to GAPDH levels. **E, F** Cellular *O*-GlcNAcylation levels were regulated by **(E)** 2 μM Thiamet-G (TMG) (*n*=3) or **(F)** 30 μM OSMI-1 (*n*=4) treatment in A549 cells. The cell lysates were immunoblotted with the indicated antibodies. XRCC4 expression were normalized to GAPDH levels. **G** MCF7 cells were cultured in media containing 5 or 25 mM glucose for 24 hours and transfected with control vector or OGT for 24 hours. XRCC4 expression was normalized to GAPDH levels (*n*=4). **H** A549 cells were incubated in media containing 5 or 25 mM glucose for 24 hours and transfected with control vector or OGT for 24 hours. XRCC4 expression were normalized to GAPDH levels (*n*=4). **A-H** Data are represented as means ± SD. Statistical significance was determined by the two-tailed Student's *t* test or one-way ANOVA for multiple comparisons (**P* < 0.05, ***P* < 0.01, ****P* < 0.001)

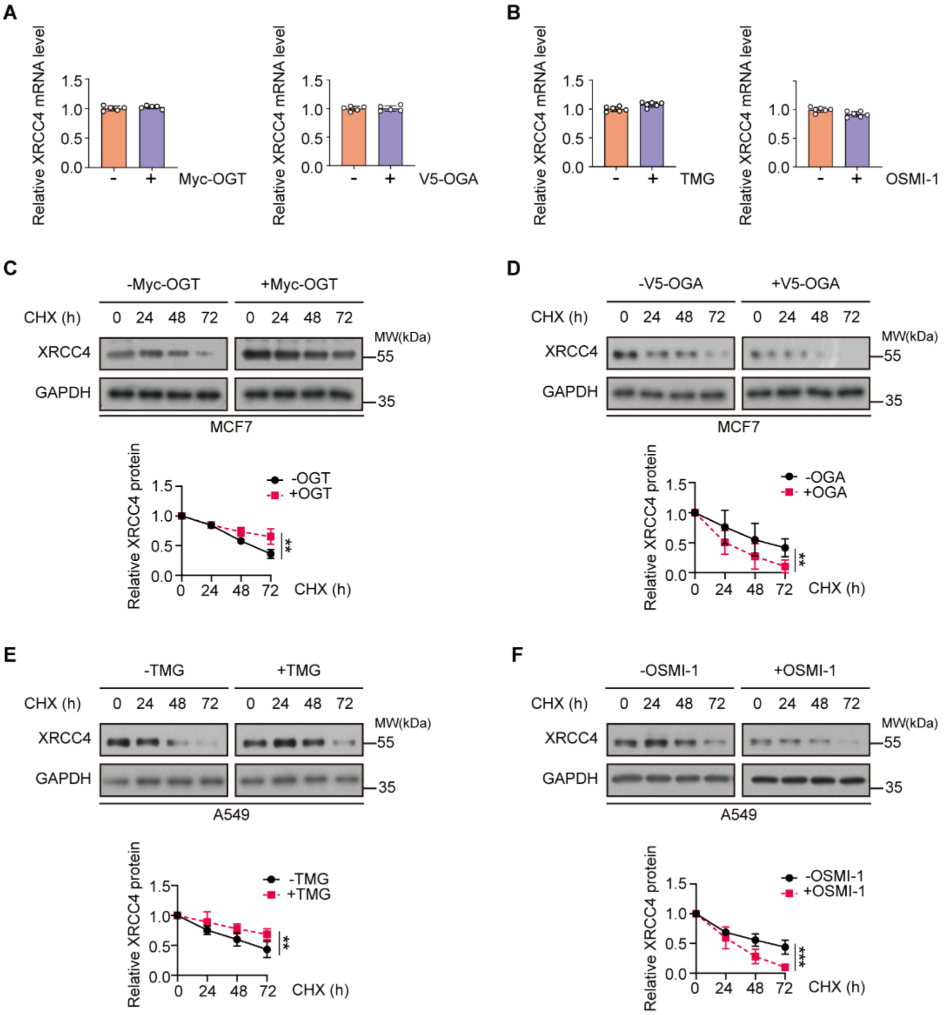

Figure S2. XRCC4 protein levels are altered in response to cellular *O*-GlcNAcylation levels in cancer cells.

A Total RNA was extracted from MCF7 cells transfected with OGT or OGA for 24 hours. XRCC4 mRNA levels were quantified by RT-qPCR and normalized to β-actin levels (*n*=5). **B** Total RNA was extracted from A549 cells treated with TMG or OSMI-1 for 24 hours. XRCC4 mRNA levels were quantified by RT-qPCR and normalized to β-actin levels (*n*=6). **C, D** MCF7 cells were transfected with **(C)** OGT (*n*=5) or **(D)** OGA (*n*=5) for 24 hours, followed by 200 μg/mL cycloheximide (CHX) treatment. Cells were collected every 24 hours. XRCC4 degradation rates were assessed by immunoblotting. XRCC4 expression was normalized to GAPDH levels. **E, F** A549 cells were treated with **(E)** TMG (*n*=4) or **(F)** OSMI-1 (*n*=5) for 24 hours, followed by 200 μg/mL cycloheximide (CHX) treatment. Cells were collected every 24 hours and the cell lysates were immunoblotted to assess the degradation rate of XRCC4. XRCC4 expression were normalized to GAPDH levels. **A-F** Data are represented as means ± SD. Statistical significance was determined by the two-tailed Student's *t* test (***P* < 0.01, ****P* < 0.001)

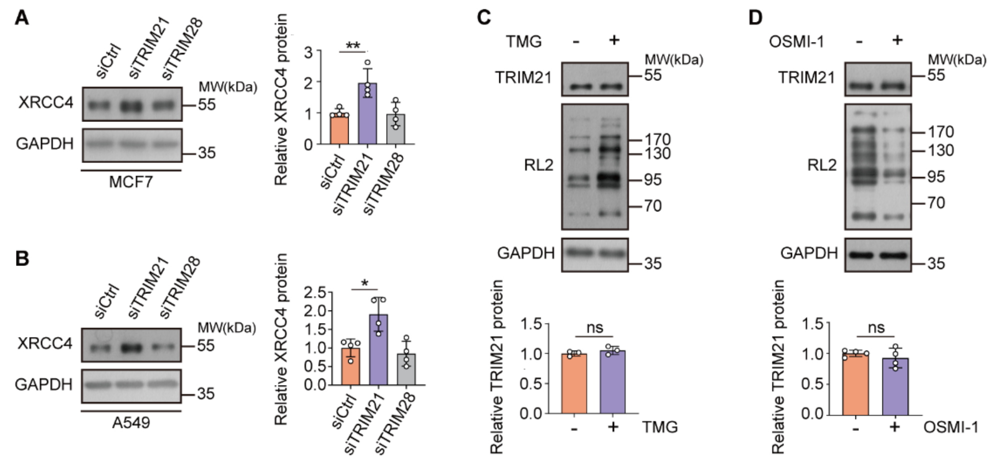

Figure S3. TRIM21 is the E3 ligase of XRCC4.

A MCF7 cells were transfected with TRIM28 or TRIM21 siRNAs for 48 hours. The cell lysates were immunoblotted with the indicated antibodies. XRCC4 expression was normalized to GAPDH levels (n=4). **B** A549 cells were transfected with TRIM28 or TRIM21 siRNAs for 48 hours. The cell lysates were immunoblotted with the indicated antibodies. XRCC4 expression was normalized to GAPDH levels (n=4). **C, D** Cellular *O*-GlcNAcylation levels were regulated by **(C)** 2 μM Thiamet-G (TMG) treatment (n=3) or **(D)** 30 μM OSMI-1 treatment (n=4). The cell lysates were subjected to immunoblotting with the indicated antibodies. TRIM21 expression was normalized to GAPDH levels. **A-D** Data are represented as means ± SD. Statistical significance was determined by the two-tailed Student's t test or one-way ANOVA for multiple comparisons (*$P < 0.05$, **$P < 0.01$, **ns** not significant)

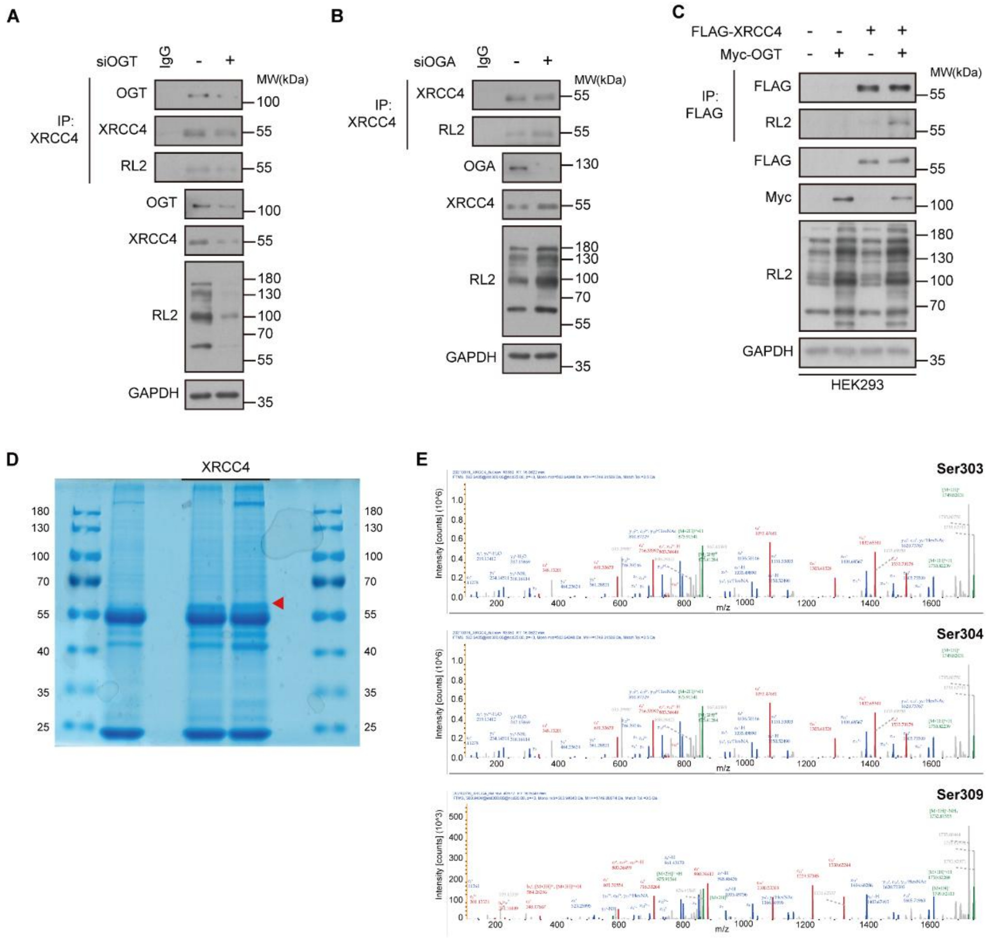

Figure S4. Identification of the major *O*-GlcNAcylation site of XRCC4.

A, B HCT116 cells were transfected with **(A)** OGT siRNA or **(B)** OGA siRNA for 48 hours. The cell lysates immunoprecipitated using XRCC4 antibody. Endogenous *O*-GlcNAcylation of XRCC4 was detected by immunoblotting with RL2 antibody. **(A)** For co-immunoprecipitation, the immunoprecipitated lysates were immunoblotted with OGT antibody. **C** HEK293 cells were transfected with FLAG-XRCC4 or Myc-OGT for 24 hours. The cell lysates were immunoprecipitated using FLAG beads. Exogenous *O*-GlcNAcylation of XRCC4 was detected by immunoblotting with RL2 antibody. **D** HEK293 cells were transfected with FLAG-XRCC4 and treated with TMG for 24 hours. The cell lysates were subjected to immunoprecipitation with FLAG-beads. FLAG-XRCC4 were detected by Coomassie brilliant blue staining. **E** Identification of putative *O*-GlcNAc sites via mass spectrometry analysis. EThcD spectra of *O*-GlcNAcylated XRCC4 peptides, 297-ENSRPDSSLPEtSK-310, is shown. MS analysis identified Ser303, Ser304, Thr308 (Thr308 EThcD spectra is presented in Figure 4D) and Ser309 as *O*-GlcNAcylation sites within this peptide.

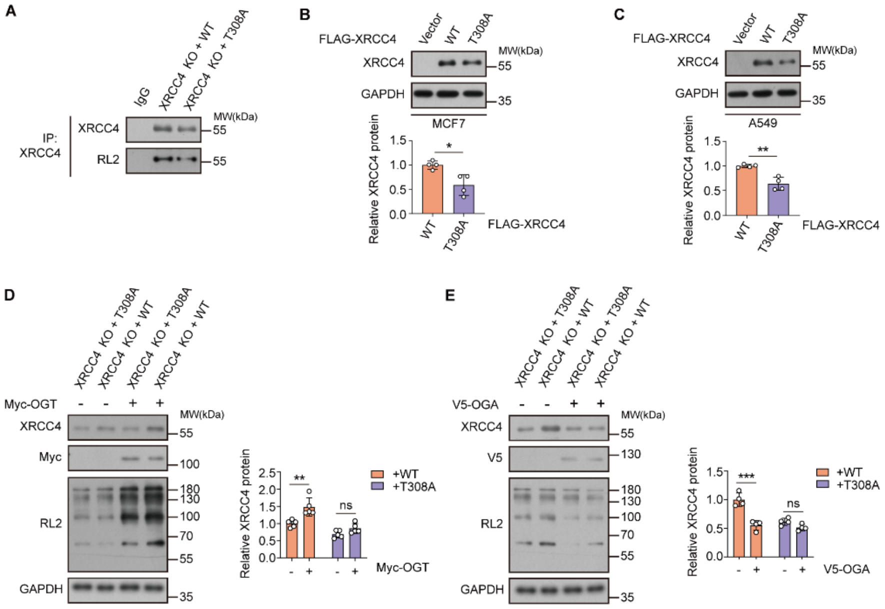

Figure S5. *O*-GlcNAc modification of XRCC4 at Thr308 controls its stability in cancer cells.

A The cell lysates from HCT116 XRCC4 KO cells stably expressing WT or T308A immunoprecipitated using XRCC4 antibody. *O*-GlcNAcylation of XRCC4 was detected by immunoblotting with RL2 antibody. **B** FLAG-XRCC4 WT or T308A were stably expressed in MCF7 XRCC4 KO cells. The cell lysates were immunoblotted with the indicated antibodies. XRCC4 expression was normalized to GAPDH levels (n=4). **C** FLAG-XRCC4 WT or T308A were stably expressed in A549 XRCC4 KO cells. The cell lysates were immunoblotted with the indicated antibodies. XRCC4 expression was normalized to GAPDH levels (n=4). **D, E** HCT116 XRCC4 KO cells stably expressing WT or T308A transfected with **(D)** Myc-OGT (n=5) or **(E)** V5-OGA (n=4) for 24 hours. The cell lysates were immunoblotted with the indicated antibodies. XRCC4 expression was normalized to GAPDH levels. **B-E** Data are represented as means ± SD. Statistical significance was determined by the two-tailed Student's t test (*$P < 0.05$, **$P < 0.01$, ***$P < 0.001$, **ns** not significant)

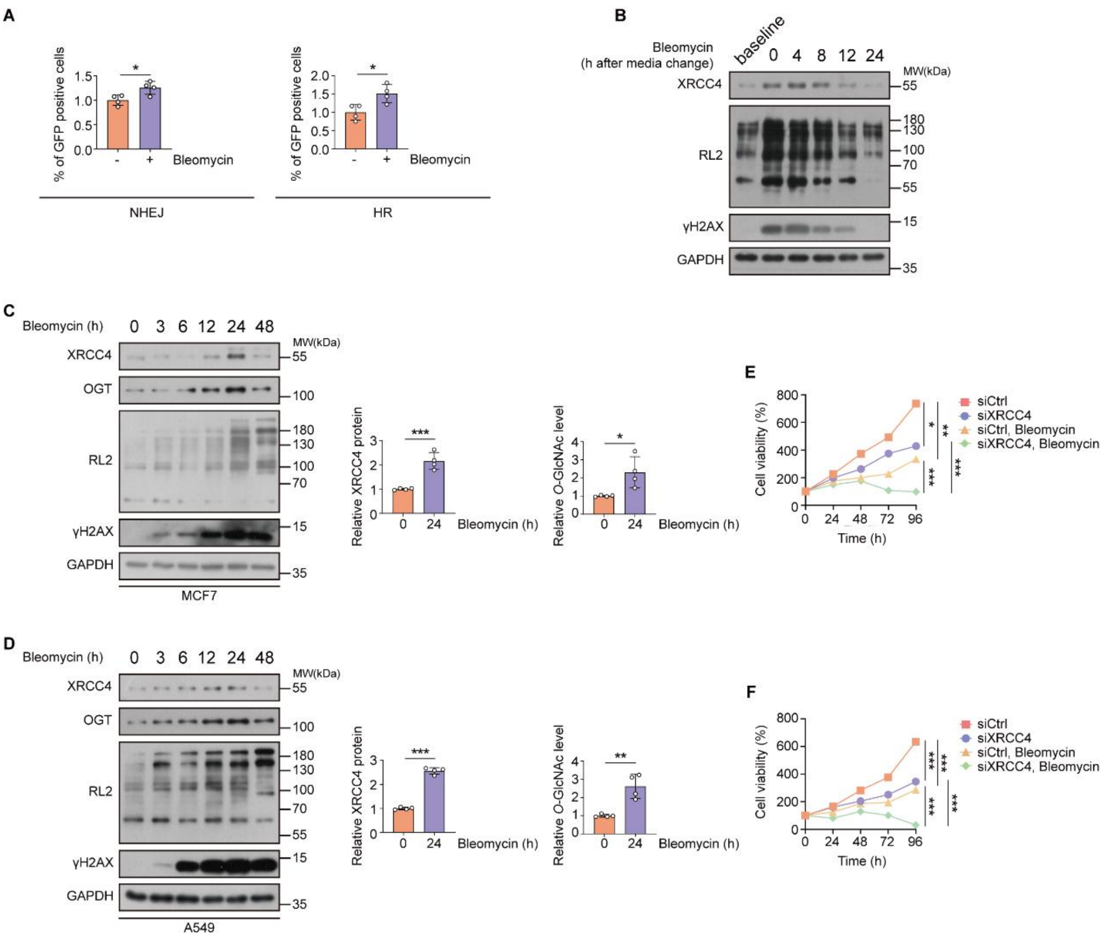

Figure S6. Cellular *O*-GlcNAcylation levels protect cancer cells against cell death in response to DNA damage.

A HeLa cells stably expressing NHEJ (EJ5-GFP) (*n*=4) or HR (DR-GFP) (*n*=4) reporter plasmids were transfected with I-SceI expression vector and treated with 10 μg/mL bleomycin for 48 h. GFP-positive cells were quantified by flow cytometry. **B** HCT116 cells were treated with 10 μg/mL bleomycin for 2 h, followed by media change to remove bleomycin. Cells were collected with the indicated time. The cell lysates were immunoblotted with the indicated antibodies. **C** MCF7 cells were treated with 10 μg/mL bleomycin. Cells were collected with the indicated time. The cell lysates were immunoblotted with the indicated antibodies. XRCC4 and *O*-GlcNAcylation levels were normalized to GAPDH levels (*n*=4). **D** A549 cells were treated with 10 μg/mL bleomycin. Cells were collected with the indicated time. The cell lysates were immunoblotted with the indicated antibodies. XRCC4 and *O*-GlcNAcylation levels were normalized to GAPDH levels (*n*=4). **E** XRCC4 was knocked down using siRNA and treated with 10 μg/mL bleomycin in MCF7 cells. WST-8 assay was performed after treatment of bleomycin for the indicated time (*n*=4). **F** XRCC4 was knocked down using siRNA and treated with 10 μg/mL bleomycin in A549 cells. WST-8 assay was performed after treatment of bleomycin for the indicated time (*n*=5). **A, C-F** Data are represented as means ± SD. Statistical significance was determined by the two-tailed Student's *t* test (**P* < 0.05, ***P* < 0.01, ****P* < 0.001)

A

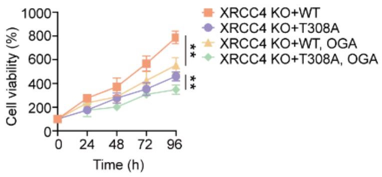

B

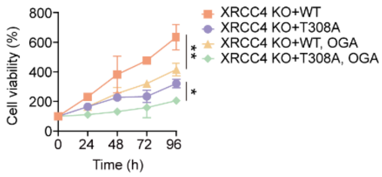

Figure S7. Cellular *O*-GlcNAcylation levels affect cancer cell viability.

A MCF7 XRCC4 KO cells stably expressing WT or T308A XRCC4 were transfected with either control vector or OGA. WST-8 assay was performed for the indicated time (*n*=5). **B** A549 XRCC4 KO cells stably expressing WT or T308A XRCC4 were transfected with either control vector or OGA. WST-8 assay was performed for the indicated time (*n*=5). **A, B** Data are represented as means ± SD. Statistical significance was determined by the two-tailed Student's *t* test or one-way ANOVA for multiple comparisons (*$P < 0.05$, **$P < 0.01$)

	SOURCE	IDENTIFIER
Rabbit polyclonal anti-Flag	MBL	Cat # PM020
Mouse monoclonal anti-GAPDH	Santa Cruz Biotechnology	Cat # sc-32233
Rabbit polyclonal anti-K48-Ub	Cell Signaling Technology	Cat # 4289
Rabbit polyclonal anti-OGT (DM-17)	Sigma-Aldrich	Cat # O6264
Mouse monoclonal anti-c-Myc	Santa Cruz Biotechnology	Cat # sc-40
Mouse monoclonal anti-V5	MBL	Cat # M167-3
Rabbit polyclonal anti-XRCC4	Santa Cruz Biotechnology	Cat # sc-271087
Mouse monoclonal anti-XRCC4	Proteintech	Cat # 15817-1-AP
Mouse monoclonal anti-RL2 (*O*-GlcNAc)	Invitrogen	Cat # MA1-072
Mouse monoclonal anti-TRIM21	Santa Cruz Biotechnology	Cat # sc-25351
Mouse monoclonal anti-phospho-H2AX	Sigma-Aldrich	Cat # 05-636-25UG
DDDDK-tagged Protein PURIFICATION GEL	MBL	Cat # 3328R
Protein A/G PLUS-Agarose	Santa Cruz Biotechnology	Cat # sc-2003
Normal Mouse IgG	Santa Cruz Biotechnology	Cat # sc-2025
Normal Rabbit IgG	Cell Signaling Technology	Cat # 2729
Peroxidase AffiniPure™ Goat Anti-Mouse IgG (H + L) Secondary Antibody	Jackson ImmunoResearch Inc.	Cat # 115-035-003
Peroxidase AffiniPure™ Goat Anti-Rabbit IgG (H + L) Secondary Antibody	Jackson ImmunoResearch Inc.	Cat # 111-035-003
Peroxidase AffiniPure™ Goat Anti-Mouse IgG, Light Chain Specific Secondary Antibody	Jackson ImmunoResearch Inc.	Cat # 115-035-174

Supplementary Table S1. Antibodies

Accession	Description	GN
Q13426	DNA repair protein XRCC4	XRCC4
P11142	Heat shock cognate 71 kDa protein	HSPA8
P0DMV8	Heat shock 70 kDa protein 1A	HSPA1A
P0DMV9	Heat shock 70 kDa protein 1B	HSPA1B
P22061	Protein-L-isoaspartate(D-aspartate) O-methyltransferase	PCMT1
P52272	Heterogeneous nuclear ribonucleoprotein M	HNRNPM
P17066	Heat shock 70 kDa protein 6	HSPA6
P01023	Alpha-2-macroglobulin	A2M
P34931	Heat shock 70 kDa protein 1-like	HSPA1L
Q9P258	Protein RCC2	RCC2
P78527	DNA-dependent protein kinase catalytic subunit	PRKDC
P06748	Nucleophosmin	NPM1
P07437	Tubulin beta chain	TUBB
P54136	Arginine--tRNA ligase cytoplasmic	RARS1
P17987	T-complex protein 1 subunit alpha	TCP1
O95278	Laforin	EPM2A
P11021	Endoplasmic reticulum chaperone BiP	HSPA5
Q9H773	dCTP pyrophosphatase 1	DCTPP1
Q08211	ATP-dependent RNA helicase A	DHX9
Q15233	Non-POU domain-containing octamer-binding protein	NONO
P09874	Poly [ADP-ribose] polymerase 1	PARP1
P49368	T-complex protein 1 subunit gamma	CCT3
A0A075B6R9	Probable non-functional immunoglobulin kappa variable 2D-24	IGKV2D-24
Q92841	Probable ATP-dependent RNA helicase DDX17	DDX17
P04843	Dolichyl-diphosphooligosaccharide--protein glycosyltransferase subunit 1	RPN1
P12004	Proliferating cell nuclear antigen	PCNA
P41252	Isoleucine--tRNA ligase cytoplasmic	IARS1
P17844	Probable ATP-dependent RNA helicase DDX5	DDX5
Q13885	Tubulin beta-2A chain	TUBB2A
Q9BVA1	Tubulin beta-2B chain	TUBB2B
P62979	Ubiquitin-40S ribosomal protein S27a	RPS27A
P62249	40S ribosomal protein S16	RPS16
P62857	40S ribosomal protein S28	RPS28
Q86V81	THO complex subunit 4	ALYREF
Q15365	Poly(rC)-binding protein 1	PCBP1
P68371	Tubulin beta-4B chain	TUBB4B
Q96HS1	Serine/threonine-protein phosphatase PGAM5 mitochondrial	PGAM5
P25398	40S ribosomal protein S12	RPS12

P48643	T-complex protein 1 subunit epsilon	CCT5
O00571	ATP-dependent RNA helicase DDX3X	DDX3X
O43242	26S proteasome non-ATPase regulatory subunit 3	PSMD3
Q13509	Tubulin beta-3 chain	TUBB3
P14866	Heterogeneous nuclear ribonucleoprotein L	HNRNPL
Q5HYC2	Uncharacterized protein KIAA2026	KIAA2026
P60891	Ribose-phosphate pyrophosphokinase 1	PRPS1
P37108	Signal recognition particle 14 kDa protein	SRP14
P26599	Polypyrimidine tract-binding protein 1	PTBP1
Q9Y4I1	Unconventional myosin-Va	MYO5A
P46109	Crk-like protein	CRKL
Q6UB35	Monofunctional C1-tetrahydrofolate synthase mitochondrial	MTHFD1L
Q13263	Transcription intermediary factor 1-beta	TRIM28
P19474	E3 ubiquitin-protein ligase TRIM21	TRIM21
Q9UJS0	Calcium-binding mitochondrial carrier protein Aralar2	SLC25A13
P62826	GTP-binding nuclear protein Ran	RAN
P14868	Aspartate--tRNA ligase cytoplasmic	DARS1
P62263	40S ribosomal protein S14	RPS14
P05388	60S acidic ribosomal protein P0	RPLP0
P62333	26S proteasome regulatory subunit 10B	PSMC6
P17812	CTP synthase 1	CTPS1
Q00839	Heterogeneous nuclear ribonucleoprotein U	HNRNPU
P49419	Alpha-aminoadipic semialdehyde dehydrogenase	ALDH7A1
O75694	Nuclear pore complex protein Nup155	NUP155
Q9BRP1	Programmed cell death protein 2-like	PDCD2L
O43684	Mitotic checkpoint protein BUB3	BUB3
P49915	GMP synthase [glutamine-hydrolyzing]	GMPS
Q15366	Poly(rC)-binding protein 2	PCBP2
P00492	Hypoxanthine-guanine phosphoribosyltransferase	HPRT1
P78347	General transcription factor II-I	GTF2I
P62701	40S ribosomal protein S4 X isoform	RPS4X
O60547	GDP-mannose 4 6 dehydratase	GMDS
Q13435	Splicing factor 3B subunit 2	SF3B2
P25205	DNA replication licensing factor MCM3	MCM3
P11908	Ribose-phosphate pyrophosphokinase 2	PRPS2
O95347	Structural maintenance of chromosomes protein 2	SMC2
Q9HCC0	Methylcrotonoyl-CoA carboxylase beta chain mitochondrial	MCCC2
P33992	DNA replication licensing factor MCM5	MCM5
P60842	Eukaryotic initiation factor 4A-I	EIF4A1
P18621	60S ribosomal protein L17	RPL17

P42167	Lamina-associated polypeptide 2 isoforms beta/gamma	TMPO
P31040	Succinate dehydrogenase [ubiquinone] flavoprotein subunit mitochondrial	SDHA
Q8NBS9	Thioredoxin domain-containing protein 5	TXNDC5
Q9Y2L1	Exosome complex exonuclease RRP44	DIS3
P23246	Splicing factor proline- and glutamine-rich	SFPQ
P47756	F-actin-capping protein subunit beta	CAPZB
P11310	Medium-chain specific acyl-CoA dehydrogenase mitochondrial	ACADM
Q15717	ELAV-like protein 1	ELAVL1
P61353	60S ribosomal protein L27	RPL27
Q6P4I2	WD repeat-containing protein 73	WDR73
P82650	28S ribosomal protein S22 mitochondrial	MRPS22
Q14168	MAGUK p55 subfamily member 2	MPP2
P0DOX8	Immunoglobulin lambda-1 light chain	
P30153	Serine/threonine-protein phosphatase 2A 65 kDa regulatory subunit A alpha isoform	PPP2R1A
P43246	DNA mismatch repair protein Msh2	MSH2
P60981	Destrin	DSTN
Q9HB71	Calcyclin-binding protein	CACYBP
P62280	40S ribosomal protein S11	RPS11
P08559	Pyruvate dehydrogenase E1 component subunit alpha somatic form mitochondrial	PDHA1
O14818	Proteasome subunit alpha type-7	PSMA7
P49736	DNA replication licensing factor MCM2	MCM2
O60229	Kalirin	KALRN
Q6P1J9	Parafibromin	CDC73
Q9UQE7	Structural maintenance of chromosomes protein 3	SMC3
Q5SW79	Centrosomal protein of 170 kDa	CEP170
Q99460	26S proteasome non-ATPase regulatory subunit 1	PSMD1
P22087	rRNA 2'-O-methyltransferase fibrillarin	FBL
O60341	Lysine-specific histone demethylase 1A	KDM1A
Q96QD9	UAP56-interacting factor	FYTTD1
O43809	Cleavage and polyadenylation specificity factor subunit 5	NUDT21
Q08945	FACT complex subunit SSRP1	SSRP1
Q96NS5	Ankyrin repeat and SOCS box protein 16	ASB16
Q14683	Structural maintenance of chromosomes protein 1A	SMC1A
Q8N1G4	Leucine-rich repeat-containing protein 47	LRRC47
Q9UHV9	Prefoldin subunit 2	PFDN2
Q8WXI9	Transcriptional repressor p66-beta	GATAD2B
P18124	60S ribosomal protein L7	RPL7
P52907	F-actin-capping protein subunit alpha-1	CAPZA1

Q96L92	Sorting nexin-27	SNX27
O95816	BAG family molecular chaperone regulator 2	BAG2
Q9C0D2	Centrosomal protein of 295 kDa	CEP295
Q8WTQ4	Uncharacterized protein C16orf78	C16orf78
P49411	Elongation factor Tu mitochondrial	TUFM
P30101	Protein disulfide-isomerase A3	PDIA3
O43837	Isocitrate dehydrogenase [NAD] subunit beta mitochondrial	IDH3B
O75489	NADH dehydrogenase [ubiquinone] iron-sulfur protein 3 mitochondrial	NDUFS3
P04637	Cellular tumor antigen p53	TP53
P61513	60S ribosomal protein L37a	RPL37A
Q14232	Translation initiation factor eIF-2B subunit alpha	EIF2B1
Q9BSD7	Cancer-related nucleoside-triphosphatase	NTPCR
Q9Y3I0	RNA-splicing ligase RtcB homolog	RTCB
Q13573	SNW domain-containing protein 1	SNW1
Q9UMY1	Nucleolar protein 7	NOL7
Q9P2J5	Leucine--tRNA ligase cytoplasmic	LARS1
P35998	26S proteasome regulatory subunit 7	PSMC2
Q9NTJ3	Structural maintenance of chromosomes protein 4	SMC4

Supplementary Table S2. XRCC4 interactome.
