# Supplementary figures and images for: O-GlcNAcylation of XRCC4 controls its stability and confers resistance to DNA double-strand break damage in cancer cells
Source: Cell Death Dis. 2026 Jan 9;17(1):22. doi: 10.1038/s41419-025-08209-4 (PMC12789502; doi:10.1038/s41419-025-08209-4)

**Fig. 1**

**A**

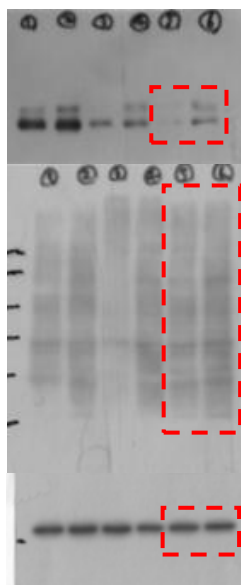

**B**

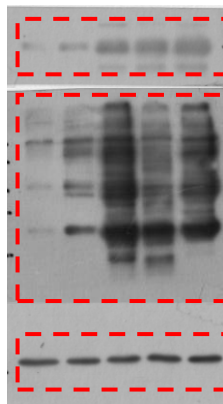

**C**

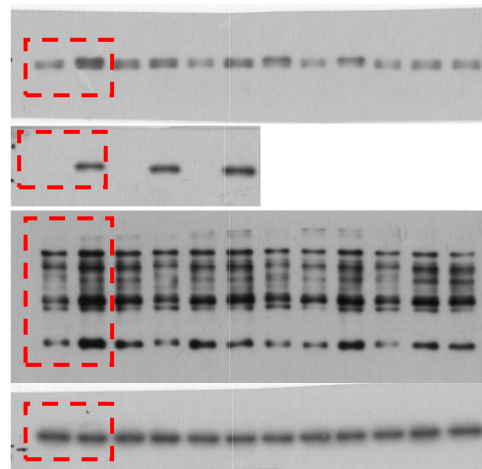

**D**

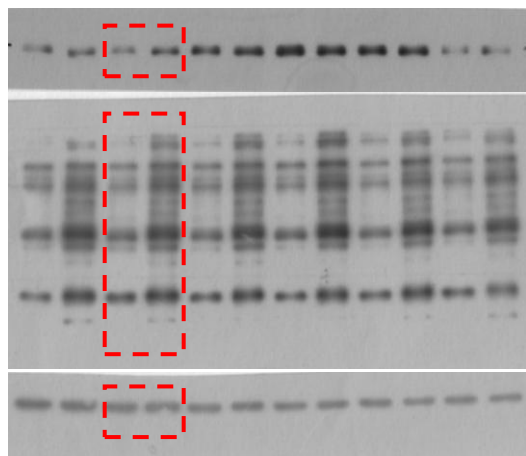

**E**

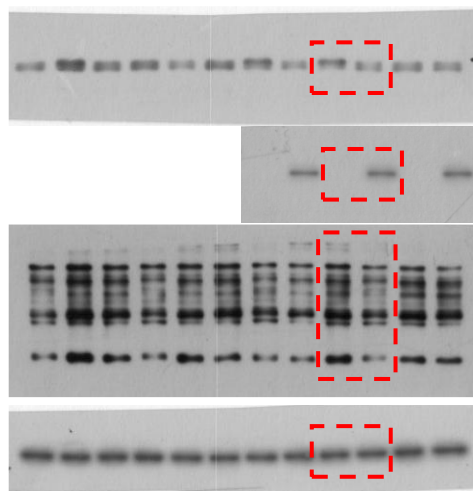

**F**

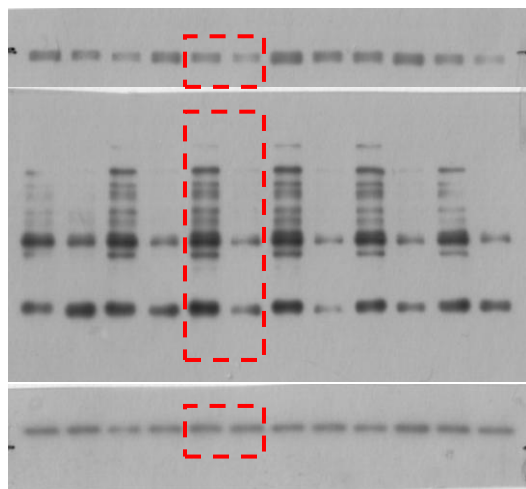

**G**

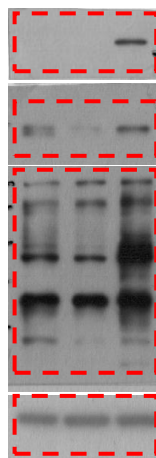

Fig. 2

B

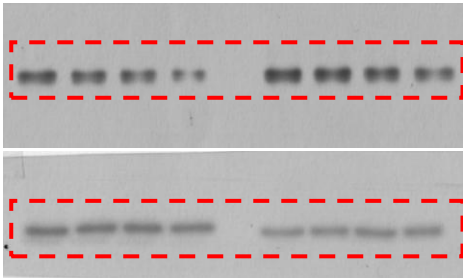

C

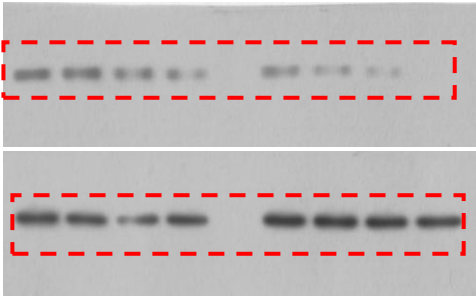

D

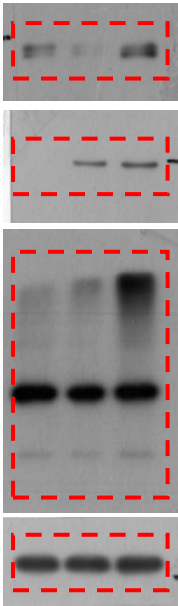

E

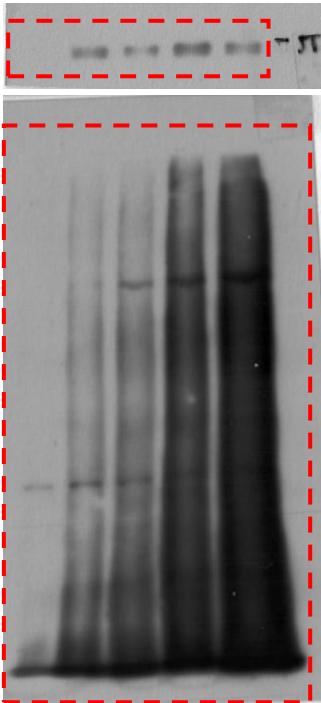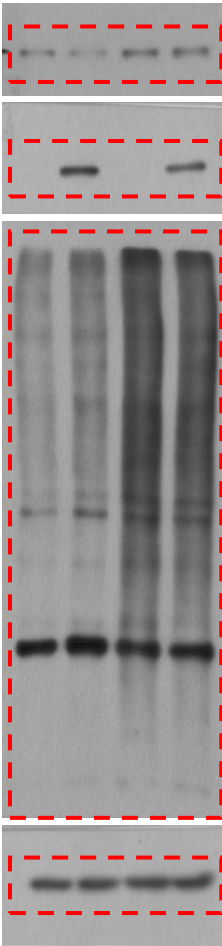

Fig. 3

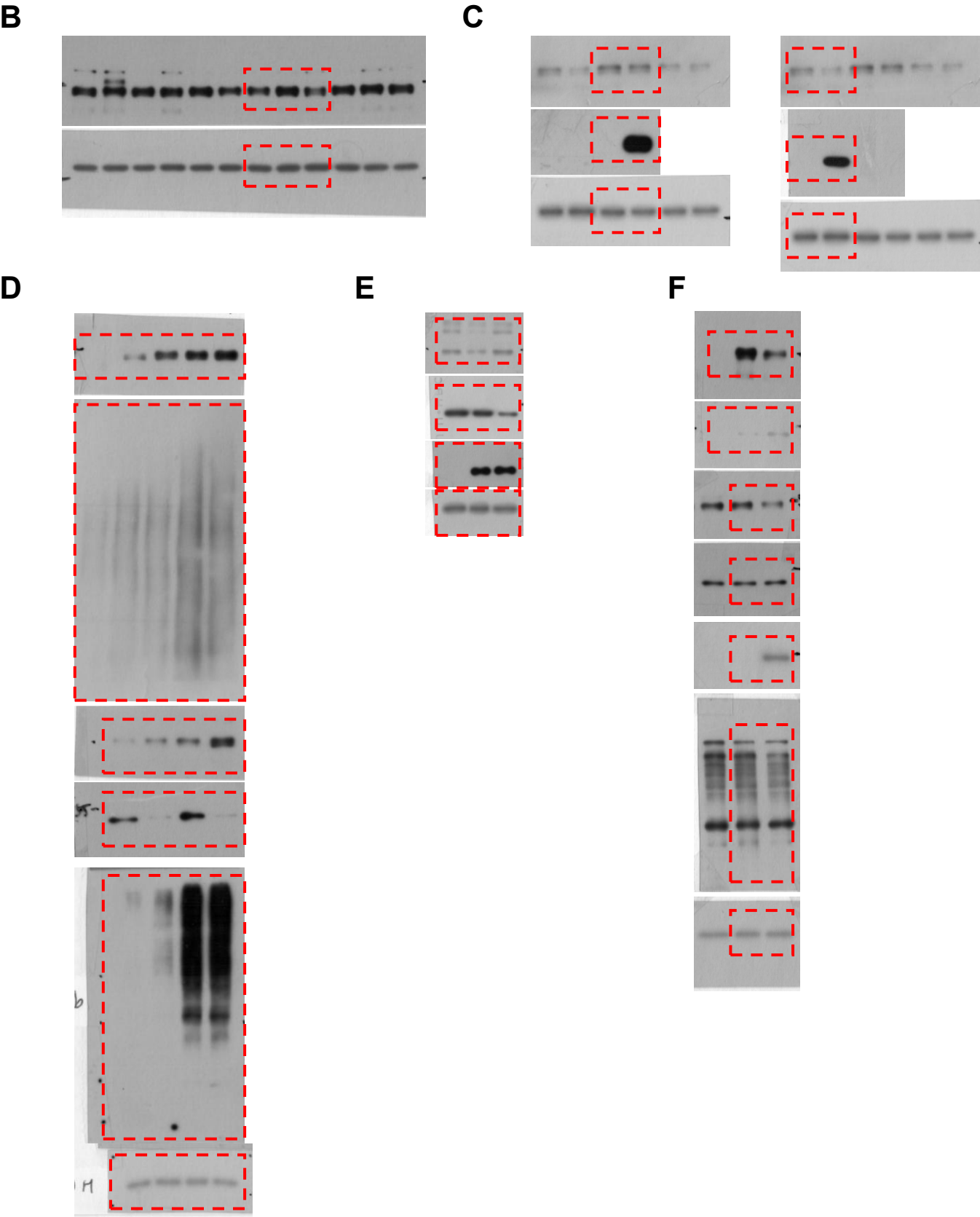

Fig. 4

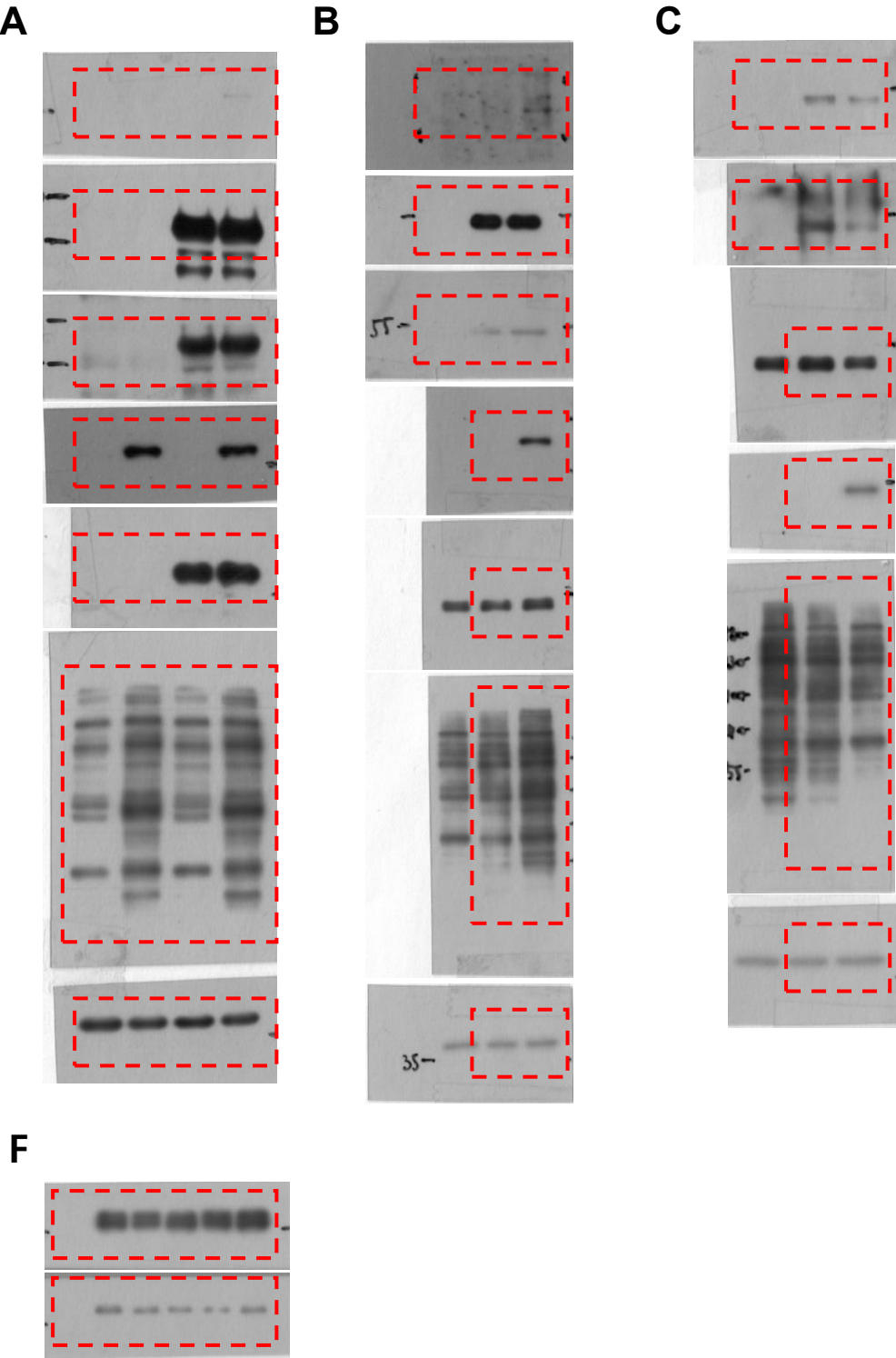

Fig. 5

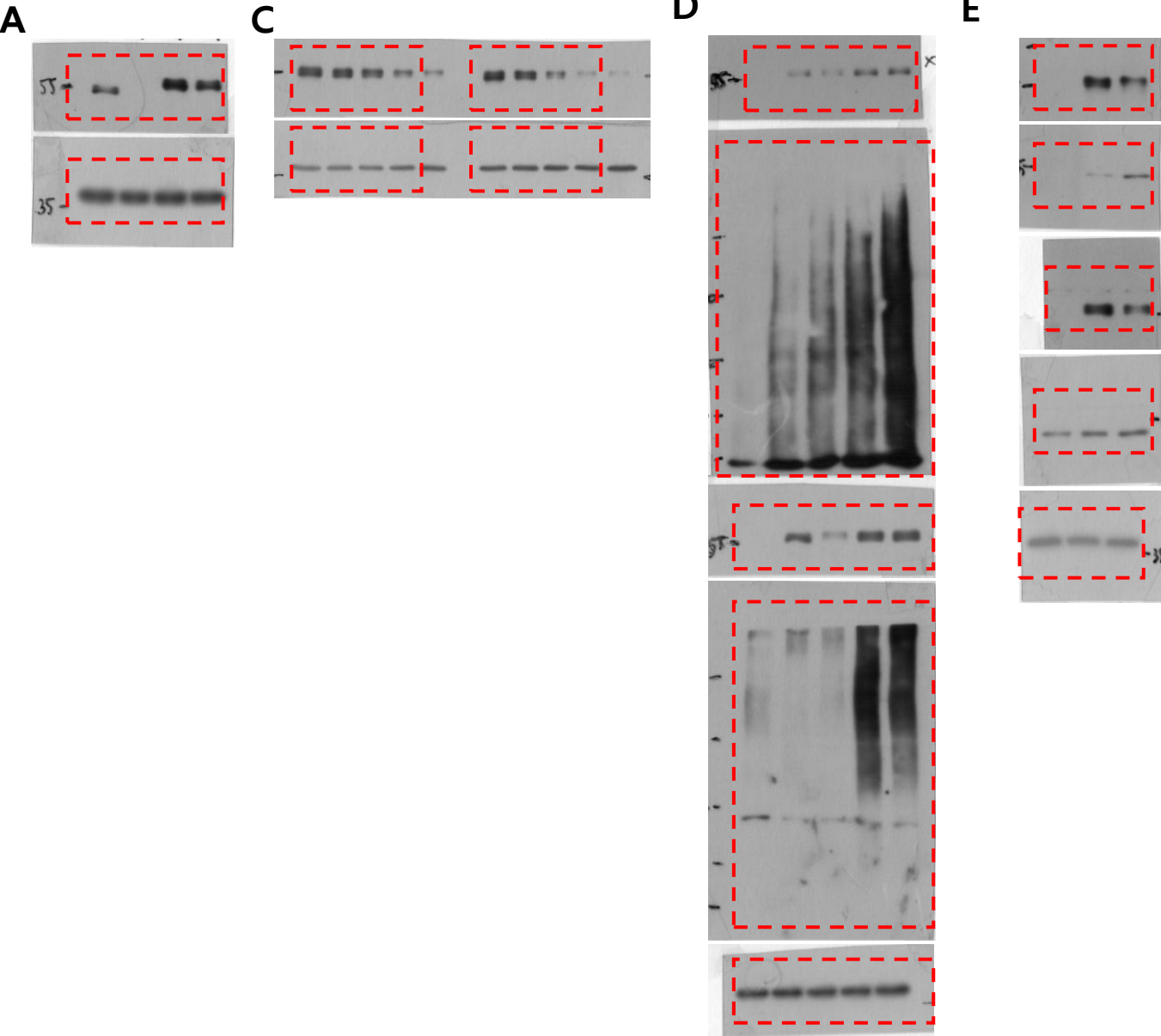

Fig. 6

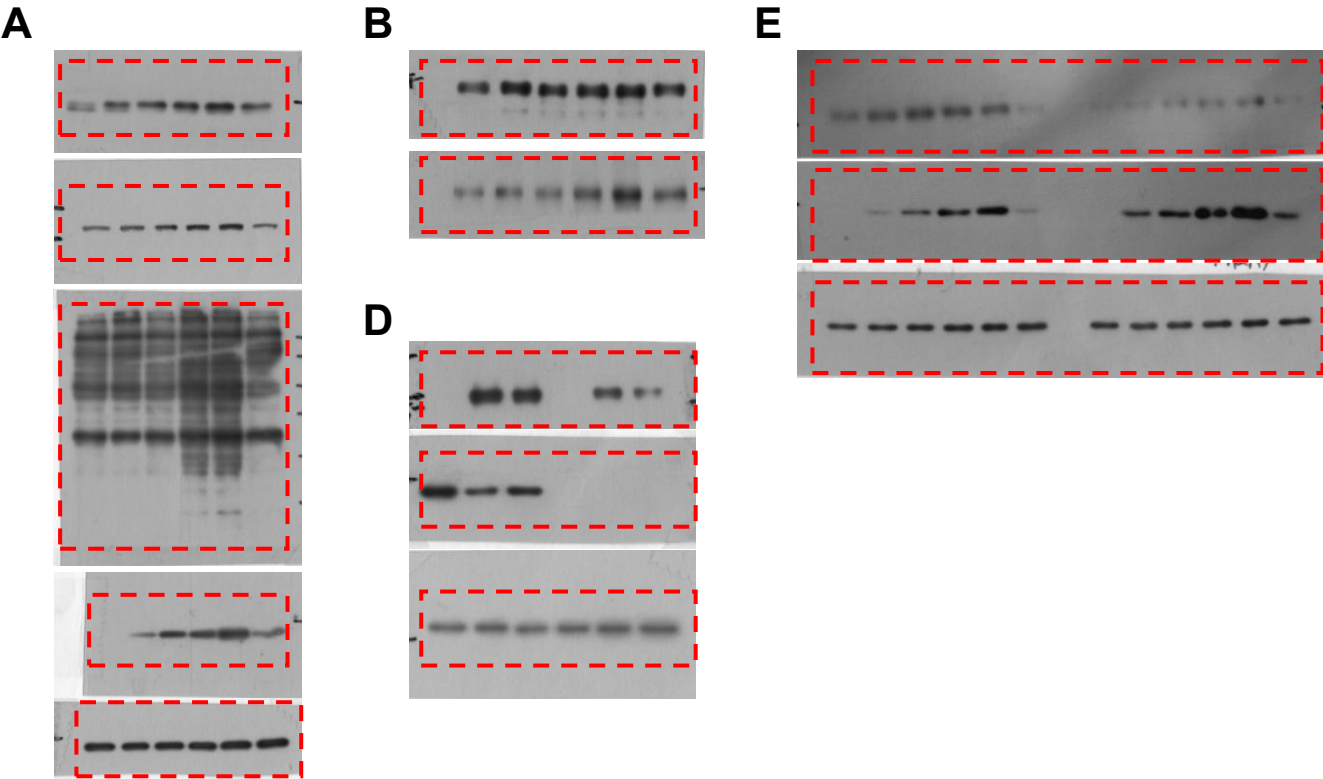

**Fig. S1**

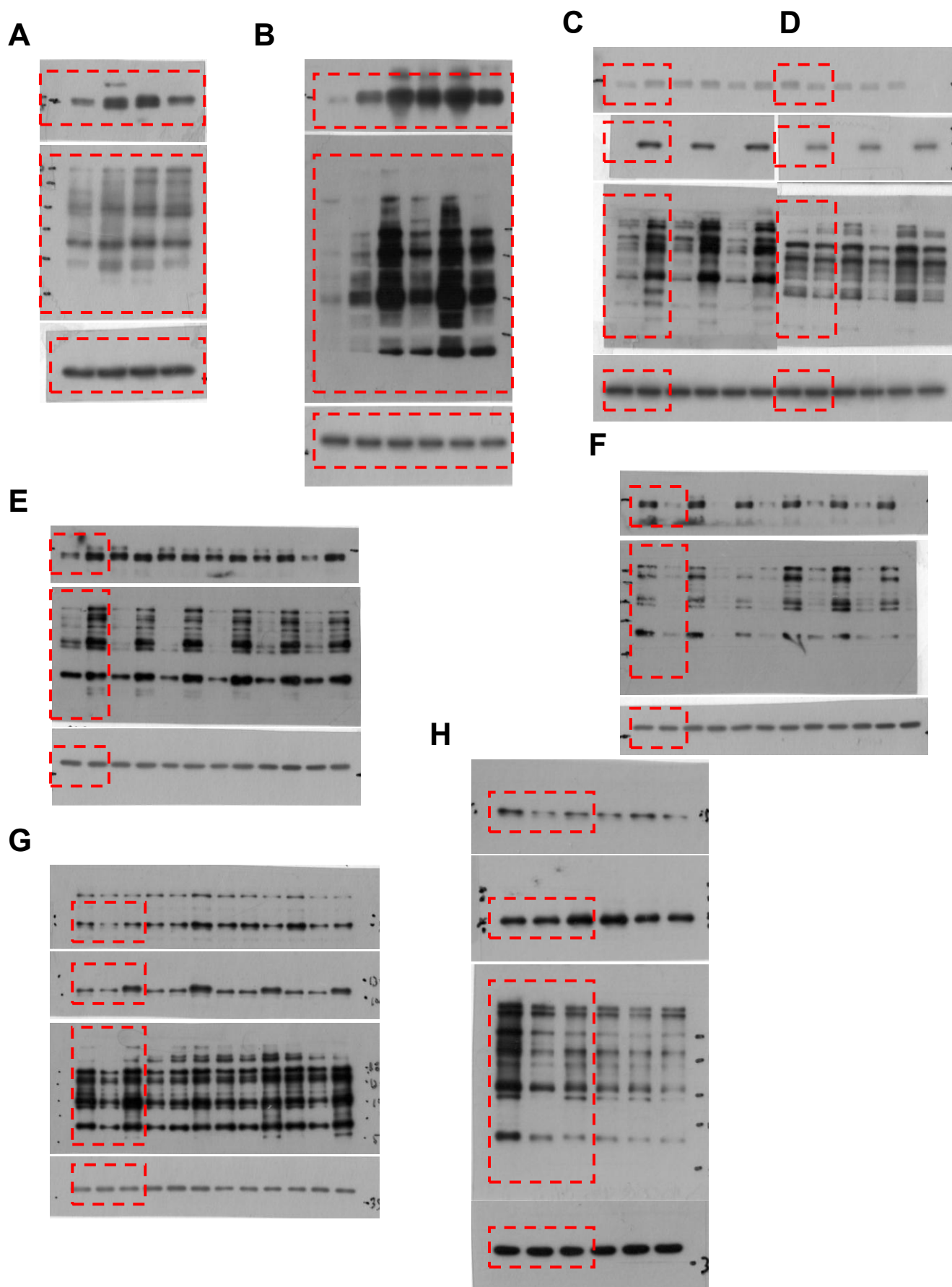

**Fig. S2**

**A**

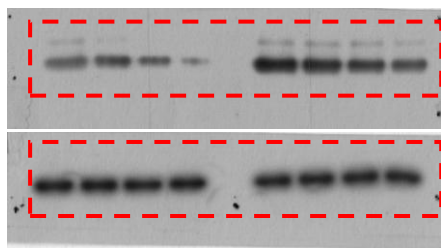

**B**

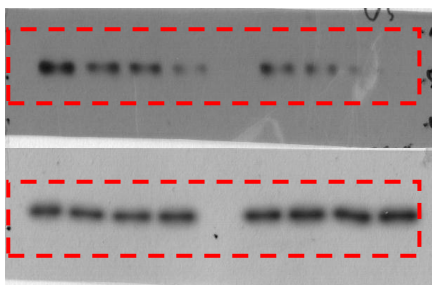

**C**

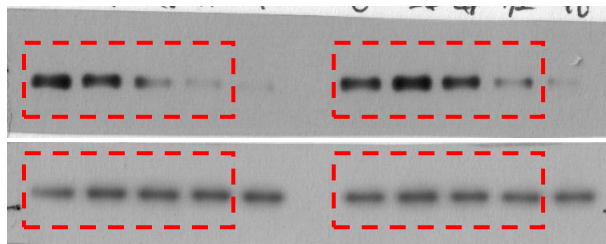

**D**

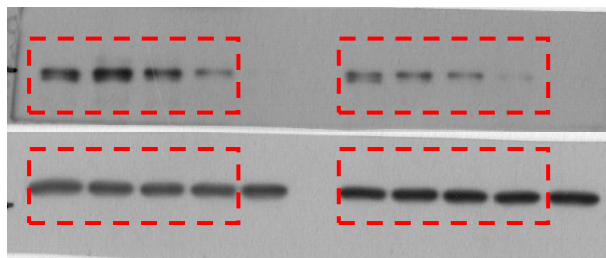

**Fig. S3**

**A**

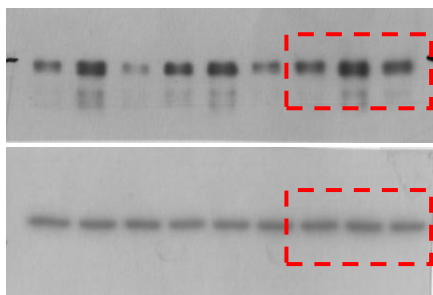

**B**

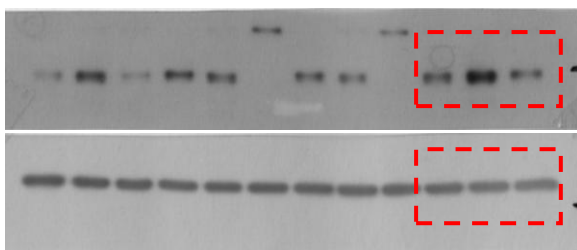

**C**

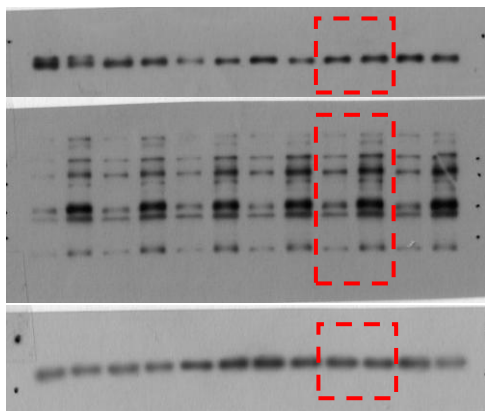

**D**

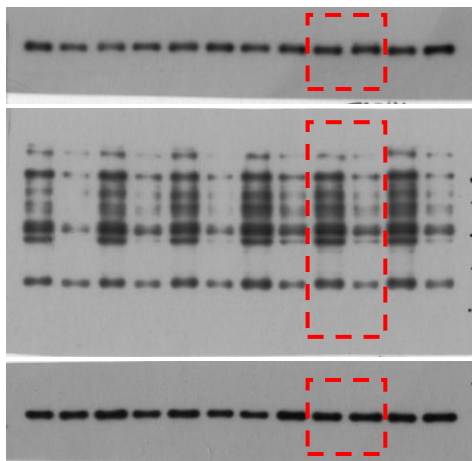

**Fig. S4**

**A**

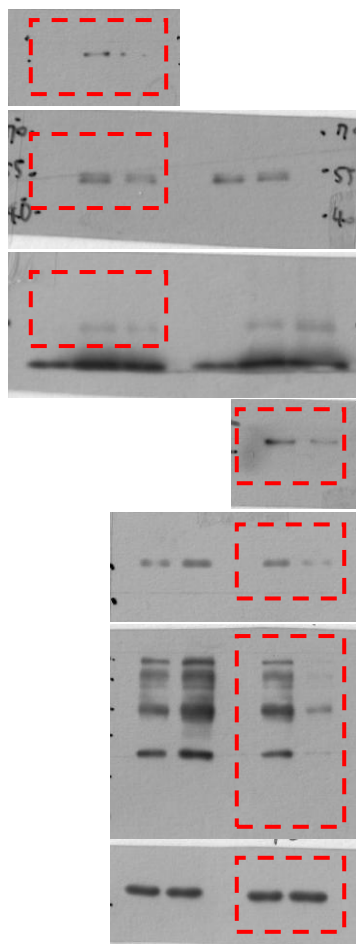

**B**

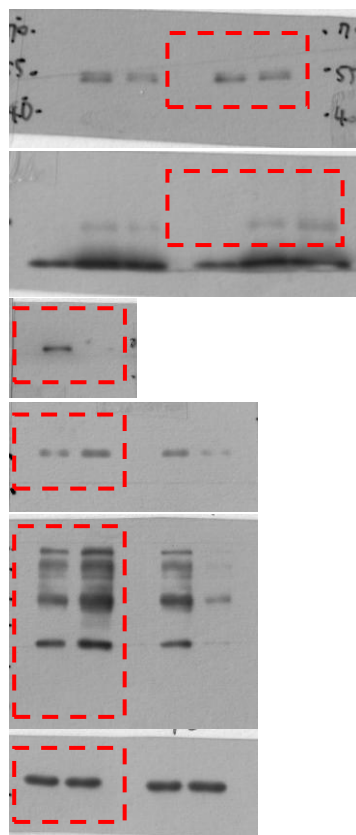

**C**

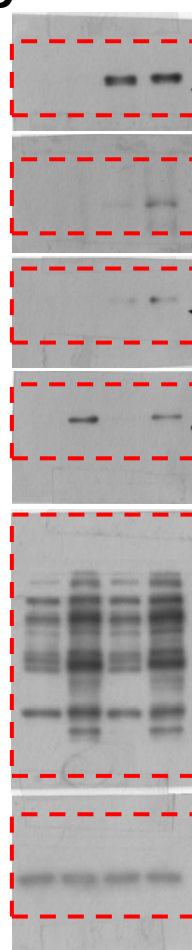

**Fig. S5**

**A**

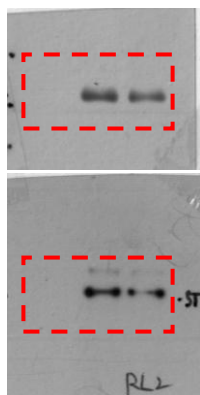

**B**

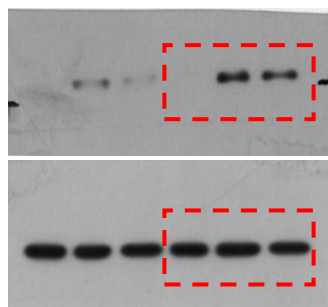

**C**

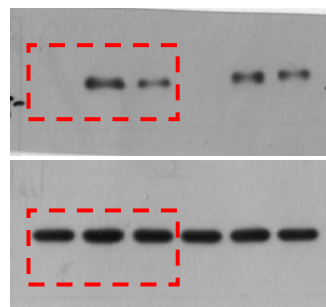

**D**

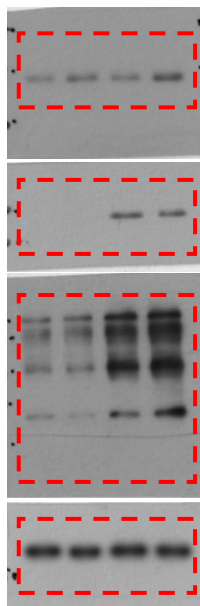

**E**

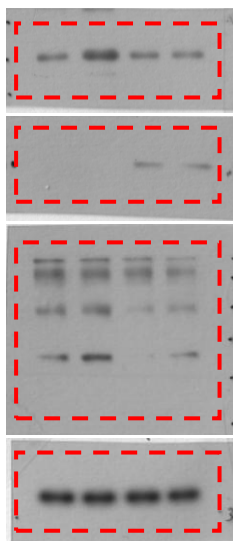

Fig. S6

B

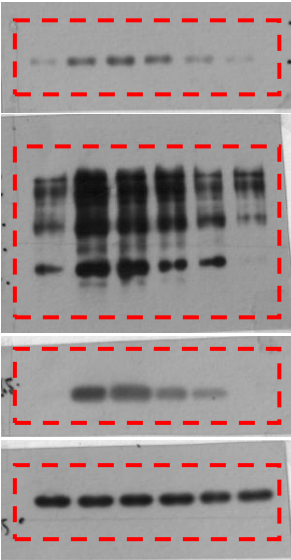

C

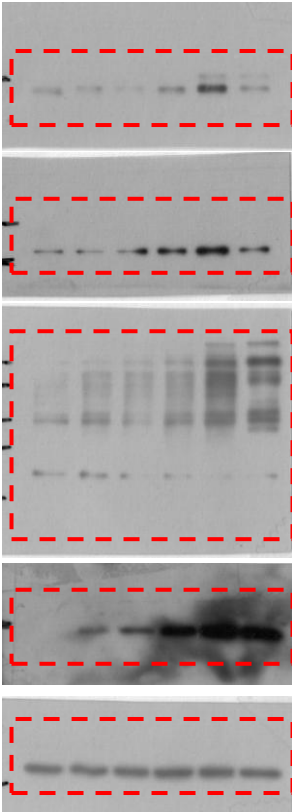

D

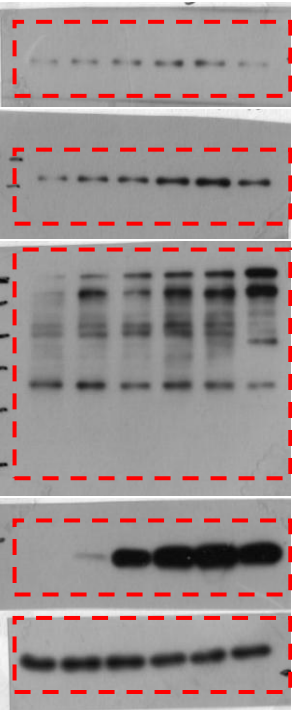

Supplement: Supplementary file 2 — Original Blot [file 41419_2025_8209_MOESM2_ESM.pdf]
